# Human heart-forming organoids recapitulate early heart and foregut development

**DOI:** 10.1038/s41587-021-00815-9

**Published:** 2021-02-08

**Authors:** Lika Drakhlis, Santoshi Biswanath, Clara-Milena Farr, Victoria Lupanow, Jana Teske, Katharina Ritzenhoff, Annika Franke, Felix Manstein, Emiliano Bolesani, Henning Kempf, Simone Liebscher, Katja Schenke-Layland, Jan Hegermann, Lena Nolte, Heiko Meyer, Jeanne de la Roche, Stefan Thiemann, Christian Wahl-Schott, Ulrich Martin, Robert Zweigerdt

**Affiliations:** 1 Leibniz Research Laboratories for Biotechnology and Artificial Organs (LEBAO), Department of Cardiothoracic, Transplantation and Vascular Surgery (HTTG), REBIRTH–Research Center for Translational Regenerative Medicine, Hannover Medical School, Hannover, Germany; 2 Department of Women’s Health, Research Institute for Women’s Health, Eberhard Karls University Tübingen, Tübingen, Germany; 3 The Natural and Medical Sciences Institute (NMI) at the University of Tübingen, Reutlingen, Germany; 4 Department of Medicine/Cardiology, Cardiovascular Research Laboratories, David Geffen School of Medicine at UCLA, Los Angeles, CA USA; 5 Cluster of Excellence iFIT (EXC 2180) ‘Image-Guided and Functionally Instructed Tumor Therapies’, Eberhard Karls University Tübingen, Tübingen, Germany; 6 Research Core Unit Electron Microscopy, Hannover Medical School, Hannover, Germany; 7 Institute of Functional and Applied Anatomy, Hannover Medical School, Hannover, Germany; 8 Industrial and Biomedical Optics Department, Laser Zentrum Hannover, Hannover, Germany; 9 Institute for Neurophysiology, Hannover Medical School, Hannover, Germany; 10 Biomedical Research in Endstage and Obstructive Lung Disease (BREATH), Member of the German Center for Lung Research (DZL), Hannover Medical School, Hannover, Germany; 11 Present Address: Stem Cell Discovery, Novo Nordisk A/S, Måløv, Denmark

**Keywords:** Pluripotent stem cells, Pattern formation

## Abstract

Organoid models of early tissue development have been produced for the intestine, brain, kidney and other organs, but similar approaches for the heart have been lacking. Here we generate complex, highly structured, three-dimensional heart-forming organoids (HFOs) by embedding human pluripotent stem cell aggregates in Matrigel followed by directed cardiac differentiation via biphasic WNT pathway modulation with small molecules. HFOs are composed of a myocardial layer lined by endocardial-like cells and surrounded by septum-transversum-like anlagen; they further contain spatially and molecularly distinct anterior versus posterior foregut endoderm tissues and a vascular network. The architecture of HFOs closely resembles aspects of early native heart anlagen before heart tube formation, which is known to require an interplay with foregut endoderm development. We apply HFOs to study genetic defects in vitro by demonstrating that *NKX2.5*-knockout HFOs show a phenotype reminiscent of cardiac malformations previously observed in transgenic mice.

## Abstract (web summary)

Heart-forming organoids model early cardiac development.

## Main

Human pluripotent stem cells (hPSCs), including embryonic stem cells (hESCs) and induced PSCs (hiPSCs), have the capability to self-organize into three-dimensional (3D) structures called organoids resembling embryo-like tissue patterns in vitro. Although organoid models have been described for a broad range of tissues^1–3^, progress in the cardiovascular field has been limited. Recent studies of cardiac microtissues or organoids have improved on prior heart muscle engineering approaches^4,5^. However, this work has focused on engineering pre-differentiated or primary cardiac cell types to mimic adult-like heart tissue^6–10^. For example, Mills et al.^7^ and Voges et al.^9^ generated organoids by embedding hPSC-derived cardiomyocytes in a collagen I–Matrigel mixture. These organoids reproduce some aspects of adult heart tissue, including stromal cells, an endothelial network and an epicardial layer, but do not reflect the processes of early heart development. Studies focusing on the morphological aspects of heart development are rare. Ma et al. generated ‘cardiac microchambers’ containing myofibroblasts at the perimeter and cardiomyocytes in the center by geometric confinement of hPSCs in micropatterns^11^. Using mouse PSCs, Andersen et al. derived ‘precardiac organoids’ forming two heart fields, which showed high similarities to their in vivo counterparts at the corresponding developmental stage^12^. However, these studies did not demonstrate the spatiotemporal patterning of early cardiogenesis, including the interplay with foregut endoderm. Our aim in the present study was to recapitulate early heart developmental patterns using hPSCs.

The heart is the first functional organ formed in the embryo. It originates from the splanchnic mesoderm, which emerges from the primitive streak during gastrulation. After precardiac mesoderm specification, progenies form the bilateral heart-forming regions on each side of the embryonic midline in close proximity and inductive exchange with the developing foregut endoderm^13^. Cells of the heart-forming regions extend across the midline and fuse into the heart tube, which consists of two layers, the myocardium and the endocardium. Later, the heart tube undergoes looping, which positions the future heart chambers. The outermost layer of the heart, the epicardium, is derived from a progenitor cell population called the ‘proepicardium’ and gives rise to cardiac fibroblasts and smooth muscle cells^14^.

We describe a robust method to reproduce the first steps of human cardiogenesis in vitro. As methods to generate organoids representing other tissues typically use encapsulation of hPSCs in Matrigel^1^, we here combine this strategy with directed cardiac differentiation by WNT pathway modulation. The resulting HFOs closely resemble early embryonic heart anlagen, including nascent foregut endoderm. Furthermore, we demonstrate an application of the model to monitor gene-knockout (KO) phenotypes in vitro.

## Results

### Robust HFO formation by differentiation of Matrigel-embedded hPSC aggregates

**Fig. 1:**
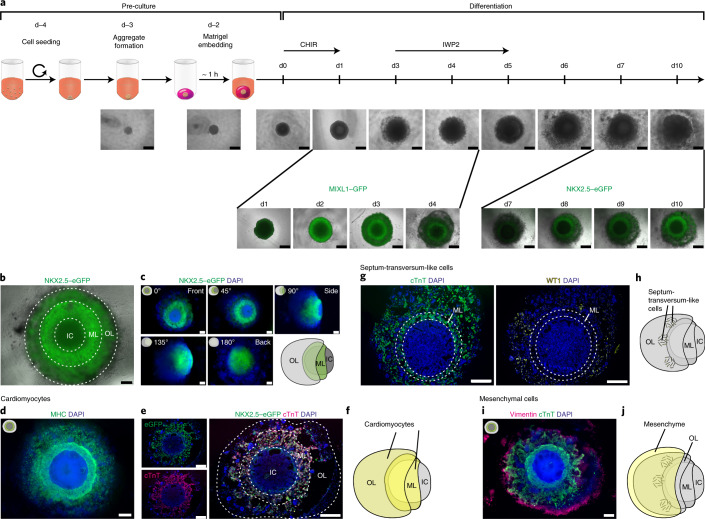
HFOs recapitulate patterns of early cardiomyogenesis. **a**, The protocol for HFO formation. hPSC aggregates were individually embedded in Matrigel and differentiated using CHIR and IWP2. The figure shows the development of HFOs from d−4 until d10 of differentiation (upper row) as well as MIXL1–GFP and NKX2.5–eGFP expression patterns (bottom row). **b**, A typical HES3 NKX2.5–eGFP-derived HFO forming three layers: IC, ML and OL. **c**, An HFO rotated around its virtual axis and respective schemes. **d**, Whole-mount IF staining for the myocardial marker MHC. **e**, A paraffin section stained for cTnT and an anti-GFP antibody. **f**, A scheme of an HFO with highlighted cardiomyocytes. **g**, Sequential paraffin sections stained for cTnT and the ST marker WT1. **h**, A scheme of an HFO with highlighted ST-like cells. **i**, Whole-mount IF staining for the mesenchymal cell marker vimentin. **j**, A scheme of an HFO with highlighted mesenchymal cells. Scale bars, 500 µm (**a**) and 200 µm (**b**–**e**,**g**,**i**).

Free-floating hPSC aggregates efficiently differentiate into highly pure cardiomyocytes in suspension culture in response to canonical WNT pathway modulation by small molecules such as CHIR99021 (CHIR; WNT pathway activator) and IWP2 (WNT pathway inhibitor)^15,16^. For HFO formation, we applied an equivalent protocol but using individual hPSC aggregates of a defined size (based on the cell number seeded for aggregate formation on differentiation day minus 4 (d−4)) embedded in Matrigel (Fig. 1a). Out of four different starting cell numbers tested (500, 3,500, 5,000 and 10,000 cells), HFO formation succeeded only with 5,000 cells (data not shown).

These modifications of our conventional cardiac differentiation protocol^15^ were inspired by studies revealing the broad range of mesendodermal lineages inducible by CHIR^17^ and by the use of Matrigel for the formation of numerous organoid types^1^. Applying a MIXL1–GFP hESC reporter line^18^, a transient GFP pattern was revealed on day 2 (d2)–d4. On d2, a GFP-negative core was surrounded by a GFP-positive ring, which was progressively covered by a GFP-negative outer layer (OL) by d4 (Fig. 1a). As MIXL1 is a mesendoderm marker transiently expressed in the primitive streak during gastrulation, these observations suggest the spatiotemporally confined primitive-streak-like mesendoderm induction in our model.

**Extended Data Fig. 1:**
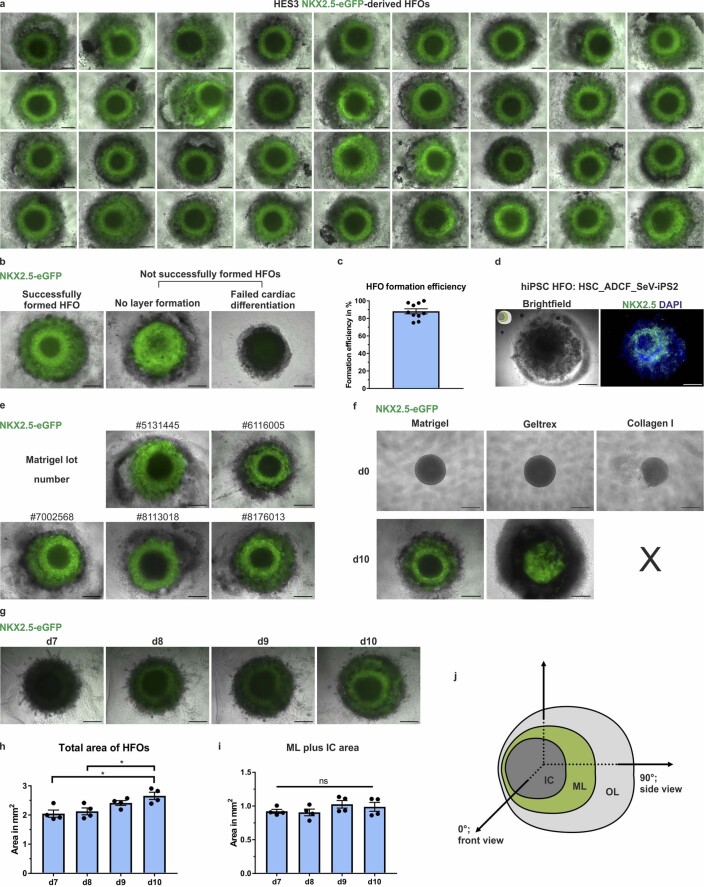
HFO formation and 3D morphology. **a**, Typical outcome of one experiment. **b**, Typical examples of successfully formed versus failed HES3 NKX2.5-eGFP-derived HFOs. Only successfully formed HFOs were used for subsequent experiments. **c**, HFO formation efficiency determined by the proportion of successfully formed HFOs from n = 10 independent experiments (418 HFOs in total). Data are presented as mean ± SEM. **d**, Whole mount immunofluorescence (IF) staining for NKX2.5 on an HFO derived from the hiPSC line HSC_ADCF_SeV-iPS2 showing a ring-like NKX2.5 pattern equivalent to HES3 NKX2.5-eGFP-derived HFOs. **e**, Representative images of HES3 NKX2.5-eGFP-derived HFOs differentiated in five different Matrigel lots. **f**, Comparison of HES3 NKX2.5-eGFP-derived HFOs differentiated in Matrigel, Geltrex or collagen I on d0 and d10 of differentiation. **g**, Representative images of HES3 NKX2.5-eGFP-derived HFOs from d7 – d10 of differentiation. **h**, Total area of HFOs from d7 – d10 of differentiation. n = 4 HFOs from one experiment; one-way ANOVA (Tukey’s multiple comparison test); data are presented as mean ± SEM; d7/d10: *P = 0.0115; d8/d10: *P = 0.0268. (i) Area of myocardial layer (ML) plus inner core (IC) from d7 – d10 of differentiation. n = 4 HFOs from one experiment; one-way ANOVA (Tukey’s multiple comparison test); data are presented as mean ± SEM; ns = not significant (P > 0.05). (j) Scheme of an HFO with defined axes. OL=outer layer. Scale bars: **a**–**g**: 500 µm.

Through progressing differentiation, organoids of about 2 mm in diameter were obtained within 14 days (Fig. 1a). Monitoring cardiomyogenesis by an NKX2.5–eGFP hESC reporter line^19^ revealed a ring-like eGFP expression pattern (reminiscent of the MIXL1–GFP pattern) of constantly increasing intensity from d7 onwards (Fig. 1a). The proportion of successfully formed HFOs with such an NKX2.5–eGFP pattern was very high across all experiments as indicated by a mean success rate of about 88% (Extended Data Fig. 1a–c). In addition to the MIXL1– and NKX2.5–eGFP hESC reporter lines, robustness of the protocol was confirmed by HFO formation from the hiPSC line HSC_ADCF_SeV-iPS2^20^ (Extended Data Fig. 1d) and by applying five different Matrigel lots, leading to unchanged morphology of the resulting organoids (Extended Data Fig. 1e). To test HFO generation with other matrices, hPSC aggregates were embedded in Geltrex (growth-factor-reduced Matrigel preparation) or collagen I (often used for cardiac tissue engineering). Using Geltrex, some cardiomyocyte formation occurred, but no layered NKX2.5–eGFP pattern was observed (Extended Data Fig. 1f). Using collagen I resulted in smaller and less dense aggregates two days after embedding (Extended Data Fig. 1f) followed by cell death. Together, these findings indicate that Matrigel is indispensable for proper HFO formation at present.

### HFOs recapitulate patterns of early cardiomyogenesis

At d10, HES3 NKX2.5–eGFP-derived HFOs consisted of a compact, NKX2.5–eGFP-positive myocardial layer (ML) enclosing an eGFP-negative inner core (IC). The ML was further covered by an OL of more loosely appearing NKX2.5–eGFP-positive and -negative cells (Fig. 1b). From d7 (upcoming NKX2.5–eGFP expression) until d10 (endpoint analysis), HFOs significantly increased in size (Extended Data Fig. 1g,h), which was related to an expansion of the OL, as the ML plus IC area remained almost constant over time (Extended Data Fig. 1i).

The layered NKX2.5–eGFP pattern was observed by fluorescence microscopy from the culture plate bottom and is hereafter defined as the front view at 0° (Extended Data Fig. 1j). Rotating HFOs around their vertical axis revealed that the ML extended into a half-shell-like structure enclosing the IC on its back side; accordingly, the OL was found to extend into a half-shell covering the back side of the ML (Fig. 1c). Scanning laser optical tomography (SLOT) supported these observations (Supplementary Video 1), corroborating the radial symmetry of HFOs.

**Extended Data Fig. 2:**
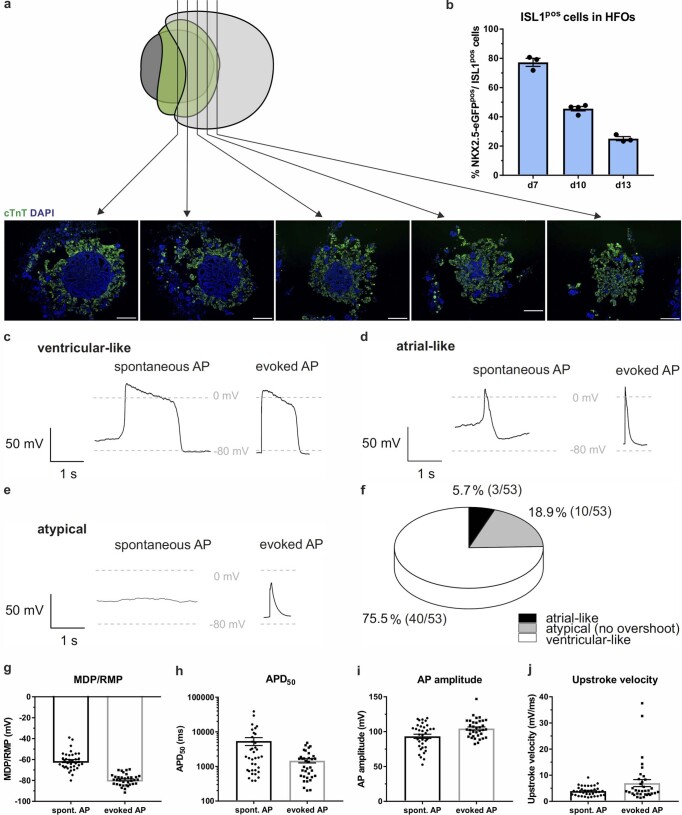
HFOs recapitulate patterns of early cardiomyogenesis. **a**, Paraffin sections from different parts of the HFO stained for cardiac troponin T (cTnT) showing that the cTnT-positive myocardial layer encloses the cTnT-negative inner core on its back side. **b**, Proportion of NKX2.5-eGFP/ ISL1-double-positive (second heart field-like) cells among all NKX2.5-eGFP-positive cells in HFOs from d7 – d13 of differentiation. n = 3 (d7, d13) or 4 (d10) derived from 3 independent experiments; data are presented as mean ± SEM. (**c**–**j**) Patch clamp analysis of HFO-derived NKX2.5-eGFP-positive cardiomyocytes in whole-cell current clamp mode. **c**–**e**, Representative traces for three different action potential (AP) phenotypes. Left panels show spontaneous electrical activity of the cells; right panels depict subsequently evoked APs of the same cell. Here, the cell membrane was hyperpolarized by application of negative holding currents (from −2 to −50 pA) to achieve comparable conditions for each cell mimicking physiological resting membrane potentials of around −80 mV. Short current pulses (1 ms, 400 – 1500 pA) were applied to evoke APs. The classification of the phenotypes was based on the AP duration of these traces: Cells were classified as ventricular-like when they showed APs with a plateau phase longer than 200 ms as measured at 50% repolarization level (APD_50_) (**c**). Cells were classified as atrial-like when they displayed a triangular shape with APD_50_ values of 20 – 200 ms (**d**). Cells showing APs without overshoot were termed atypical (**e**). **f**, Distribution of cardiac subtypes in HFOs based on the classification in **c**–**e**. (**g**–**j**) Additional AP parameters for ventricular-like cells isolated from HFOs (**g**: MDP/RMP, maximal diastolic potential/ resting membrane potential; **h**: APD_50_; **i**: AP amplitude; **j**: upstroke velocity). Spontaneous AP: n = 36 cells, evoked AP: n = 35 cells isolated from 3 HFOs from one experiment; data are presented as mean ± SEM. Scale bars: **a**: 200 µm.

The cardiomyogenic nature of the ML (and in part of the OL), indicated by NKX2.5–eGFP expression, was confirmed by immunofluorescence (IF) staining for the myocardial markers myosin heavy chain (MHC; Fig. 1d) and cardiac troponin T (cTnT; Fig. 1e,f), whereby serial frontal sections fortified the observation that the ML covers the IC on its back side (Extended Data Fig. 2a). Flow cytometry staining of HFOs from d7 to d13 for the second heart field (SHF) marker ISL1 revealed ~77% NKX2.5–eGFP/ISL1 double-positive SHF-like cells on d7, which dropped to ~25% on d13 (Extended Data Fig. 2b). In contrast, we did not detect any TBX5-positive cells representative of the first heart field (FHF), suggesting that cardiomyocytes in HFOs show an SHF-like phenotype (expected for the WNT-directed differentiation^16,21^). Patch clamping was performed to further specify the cardiac phenotype of HFO-derived NKX2.5–eGFP-positive cardiomyocytes based on their action potential characteristics. This analysis revealed that, apart from some atypical action potentials, a minor cell population (5.7%) showed an atrial-like phenotype whereas the majority (75.5%) was assigned ventricular-like (Extended Data Fig. 2c–f). For the latter, additional action potential characteristics such as the low upstroke velocity (Extended Data Fig. 2g–j) suggested an immature ventricular-like phenotype, in consistency with prior work applying chemical WNT modulation^15,16^. Initiation of HFO contraction was typically observed on d7–d10. HFOs showed distinct contraction patterns revealed by calcium imaging: typically, a circular beating pattern was found (Supplementary Video 2), but in individual HFOs, a wavefront-like propagation from a distinct initiation point was observed (Supplementary Video 3).

IF staining revealed Wilms’ tumor 1 (WT1)-positive cells predominantly located in the OL (Fig. 1g). In the embryo, WT1 is expressed by a variety of tissues, including the septum transversum (ST), hepatic stellate cells (originating from the ST-derived mesothelium lining the liver) and the proepicardium, which arises as a cluster of cells from the ST mesenchyme^14,22^. This suggests the formation of ST-like cells in the OL of HFOs (Fig. 1h). Furthermore, IF staining for vimentin revealed an outermost layer of loosely organized mesenchymal cells (Fig. 1i,j). Together, these findings show that HFOs are structured into an IC surrounded by a dense ML, which is covered by loosely arranged cardiomyocytes and ST-like cells, and ultimately enclosed by mesenchymal cells.

### CD31 staining reveals formation of VLs and endocardial-like cells

**Fig. 2:**
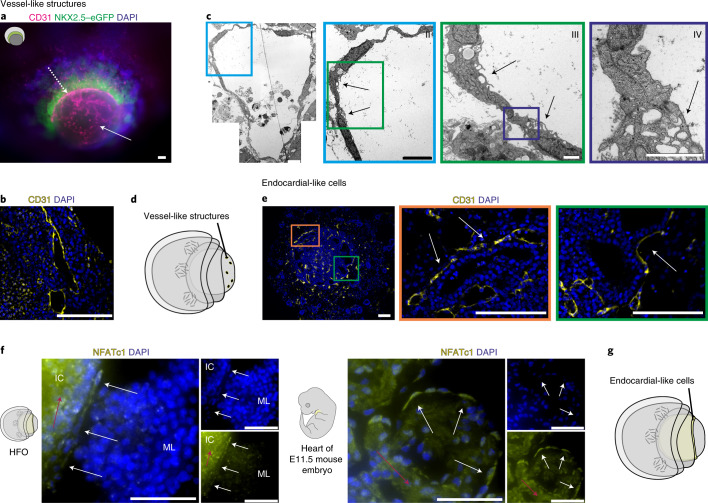
CD31 staining reveals formation of VLs and endocardial-like cells. **a**, Whole-mount IF staining for the EC marker CD31. The arrows point at ECs in the IC (solid arrow) and at the EC layer between the ML and the IC (dotted arrow). **b**, CD31 staining showing EC-lined cavities resembling VLs in the IC. **c**, TEM images of a VL in the IC with enlargements showing an endothelial junction. The arrows point at typical endothelial microvilli. **d**, A scheme of an HFO with highlighted VLs. **e**, CD31 staining with enlargements showing endocardial-like cells (arrows). **f**, Paraffin sections of an HFO (left panel) and an E11.5 mouse embryo heart (sagittal section through the ventral ventricular wall; right panel) stained for the endocardial marker NFATc1. The white arrows point at endocardial cells and the red arrows point at background staining created by the antibody. **g**, A scheme of an HFO with highlighted endocardial-like cells. Scale bars, 100 µm (**a**,**b**,**e**), 5 µm (**c**, I/II), 1 µm (**c**, III/IV) and 50 µm (**f**).

**Extended Data Fig. 3:**
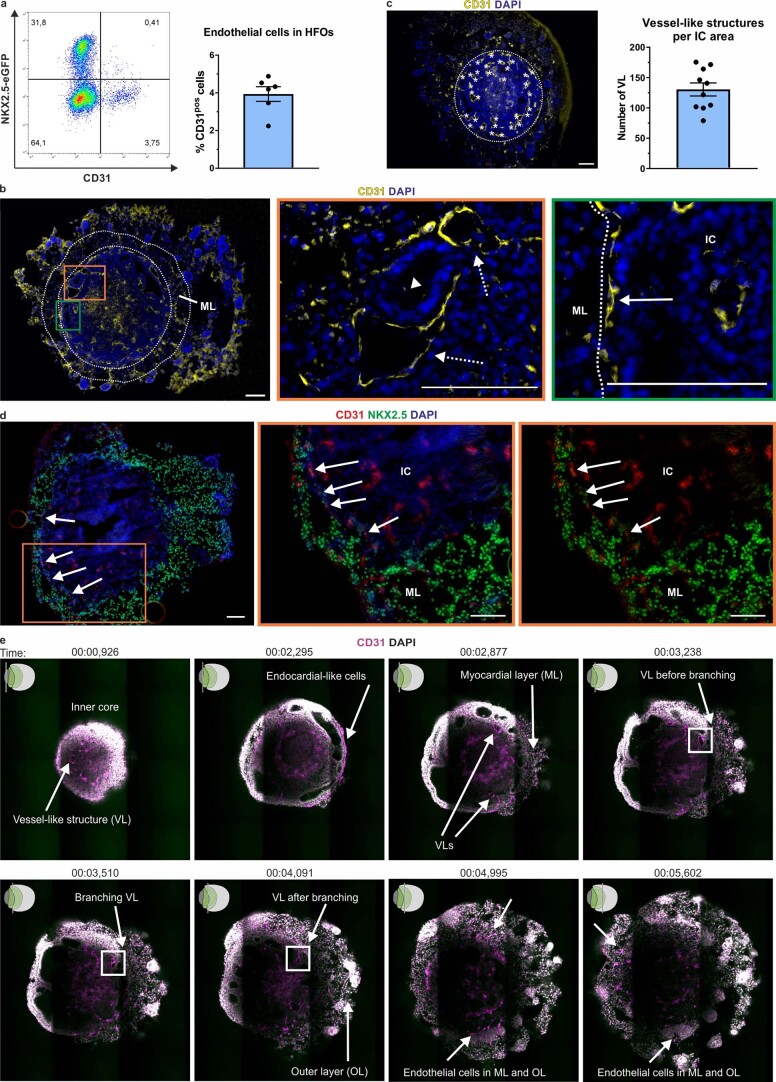
Formation of vessel-like structures and endocardial-like cells in HFOs. **a**, Representative flow cytometry plot of an HFO stained for the endothelial cell (EC) marker CD31 (left) and EC amount in HFOs assessed by flow cytometry (right). Data are presented as mean ± SEM; n = 6 HFOs from 2 independent experiments. **b**, Paraffin section stained for CD31 and enlargements showing EC-lined cavities (dotted arrows) and a non-EC-lined cavity (arrowhead) in the inner core (IC) and ECs (solid arrow) between the IC and the myocardial layer (ML). **c**, Assessment of the average number of vessel-like structures (VL) present on paraffin sections of HFOs (front view). Left: Representative image of an HFO paraffin section stained for CD31, in which the VLs are marked with asterisks. Right: Number of VLs per 1 mm² IC area. Data are presented as mean ± SEM; n = 10 HFOs from 3 independent experiments. **d**, Cryosection stained for CD31 and NKX2.5 and enlargements showing CD31/ NKX2.5-double-positive endocardial-like cells (arrows) between the ML and the IC. (**e**) Multiphoton microscopy of an HFO. The figure shows still images of Supplementary Video 4 at different time points. Scale bars: **b**–**d**: 100 µm.

Whole-mount IF staining for CD31 revealed the presence of endothelial cells (ECs) throughout the HFOs (Fig. 2a) with an average content of ~4% ECs per HFO (Extended Data Fig. 3a). These ECs formed dense, vascular-like networks particularly in the IC; in addition, a distinct EC layer lining the ML on the inside was observed (Fig. 2a). CD31 staining on paraffin sections confirmed these observations (Extended Data Fig. 3b) and highlighted that EC-lined vessel-like structures (VLs) had round or elongated cross-section profiles (Fig. 2b) whereby an average density of ~130 VLs per 1 mm^2^ IC area was observed (Extended Data Fig. 3c).

Transmission electron microscopy (TEM) further revealed EC-typical cellular junctions and microvilli-like membrane projections directed to the lumen (Fig. 2c), which is characteristic of ECs forming native blood vessels. Together, these findings substantiate our assumption that HFOs are pervaded by vessel-like anlagen particularly in the IC (Fig. 2d).

Focusing on the ECs lining the ML, we confirmed that they form a predominantly single-cell layer surrounding the IC in close proximity (Fig. 2e). In contrast to ECs forming the VL in the IC, the majority of ECs lining the ML were, in addition to CD31, co-expressing NKX2.5 (Extended Data Fig. 3d). In the embryo, at least a subset of endocardial (precursor) cells transiently express NKX2.5 in addition to endothelial markers owing to their common origin from a myocardial/endocardial precursor^23^. To test the hypothesis that these ECs indeed resemble endocardium anlagen, we performed IF staining for the endocardial marker NFATc1^24^. Testing several antibodies putatively binding to NFATc1, we found a clone showing convincing specificity while still inducing a substantial background on HFO sections as well as on embryonic day (E)11.5 mouse heart sections serving as staining controls (Fig. 2f). However, the direct comparison of NFATc1 expression on mouse heart versus HFO sections supports our assumption that the EC layer between the ML and the IC might indeed represent endocardium-like anlagen (Fig. 2f,g). Monitoring the 3D projection of ECs by multiphoton imaging of whole-mount-stained HFOs supported both assumptions—the formation of an endocardial-like layer and the presence of an extensive vascular network in the IC (Supplementary Video 4 and Extended Data Fig. 3e).

### Differential expression of SOX17, SOX2 and HNF4α reveals distinct foregut endoderm tissues

**Fig. 3:**
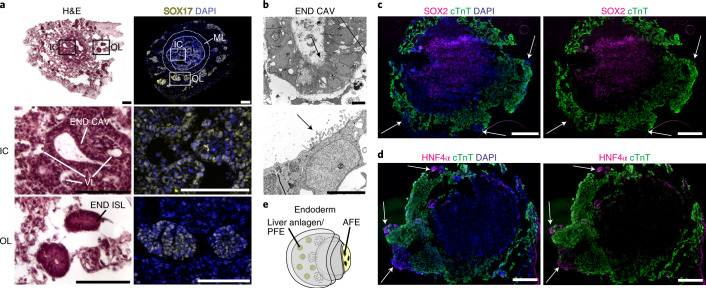
Differential expression of SOX17, SOX2 and HNF4α reveals formation of distinct foregut endoderm tissues. **a**, H&E staining (left column) and staining for the endoderm marker SOX17 (right column) with enlargements showing endodermal cavities (END CAV) in the IC and endodermal islands (END ISL) in the OL. **b**, TEM images of endodermal cavities in the IC with microvilli (arrows). **c**,**d**, Cryosections of HFOs stained for cTnT and the AFE marker SOX2 (**c**) or the hepatocyte marker HNF4α (**d**). The arrows point at endodermal islands in the OL. **e**, A scheme of an HFO with highlighted distinct endoderm tissues. Scale bars, 100 µm (**a**), 5 µm (**b**) and 200 µm (**c**,**d**).

**Extended Data Fig. 4:**
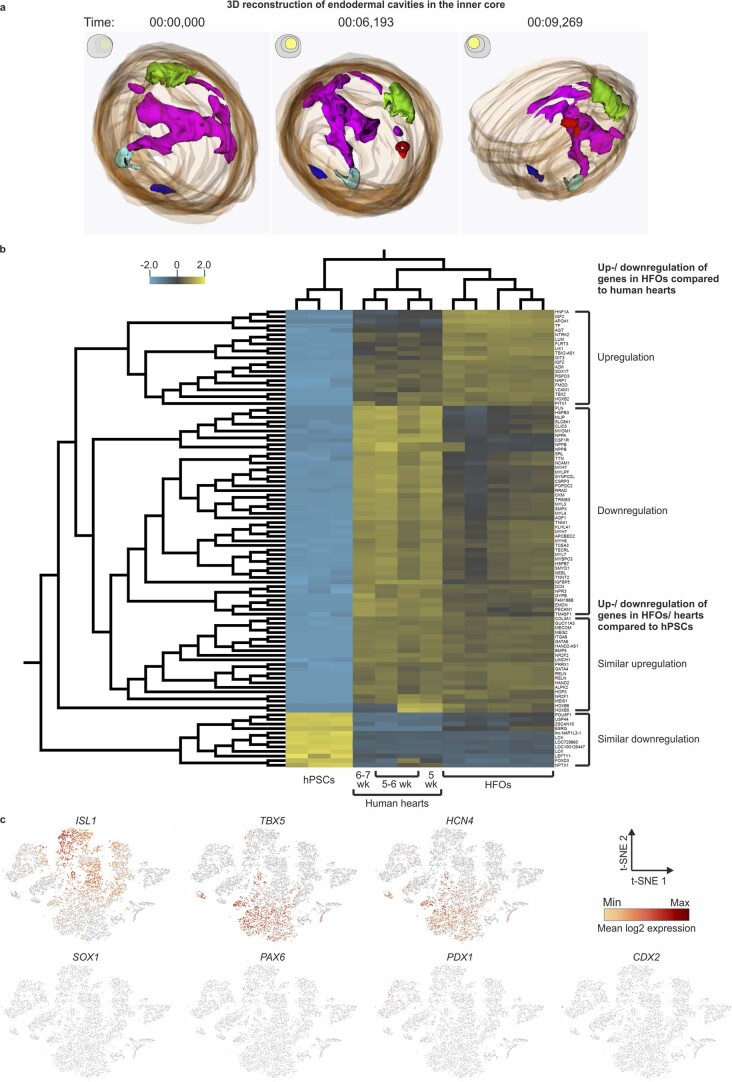
Formation of distinct foregut endoderm tissues in HFOs. **a**, 3D reconstruction of the inner core. The endodermal cavities are depicted in different colors. The figure shows still images of Supplementary Video 5 at different time points. **b**, Comparison of HFOs with human embryonic hearts. Heat map generated from the microarray data of five HFOs versus four 5–7 weeks old human embryonic hearts and three hPSC samples (q≤0.01; variance filtering = 0.5; fold change ≥ 10 to view the 100 most significantly up-/ downregulated genes). **c**, Additional t-SNE plots showing the expression of the indicated genes in two combined d13 HFOs.

In addition to EC-lined vessels, the IC harbors morphologically distinct cavities lined by brick-like epithelial cells (Fig. 3a and Extended Data Fig. 3b). Given the close developmental origin of mesendodermal derivatives, we hypothesized that this tissue might represent endoderm, as supported by positive SOX17 staining (Fig. 3a). TEM confirmed the columnar morphology of the cavity-lining epithelial cells and revealed pronounced microvilli on their apical surface directed to the cavity lumen (Fig. 3b)—a typical feature of definitive and foregut endoderm. Three-dimensional reconstruction of serial HFO sections showed that these cavities formed extensive, partially interconnected structures throughout the IC (Supplementary Video 5 and Extended Data Fig. 4a). Moreover, SOX17 staining revealed the presence of rosette-like endodermal cell islands scattered in the OL (Fig. 3a, last row). The endoderm anlagen of the IC versus the OL were not only spatially and morphologically distinct but also had specific molecular characteristics: the IC endoderm expressed SOX2 (Fig. 3c), a marker of anterior foregut endoderm (AFE) giving rise to lung, esophagus and other organs in the embryo^25^. In contrast, endodermal islands in the OL expressed HNF4α (Fig. 3d), which marks liver anlagen (derived from posterior foregut endoderm (PFE))^26^. Together, these findings demonstrate that HFOs contain distinct foregut endoderm anlagen in the IC and the OL recapitulating aspects of AFE and PFE, respectively, as schematically summarized in Fig. 3e.

### Microarray analysis and single-cell RNA sequencing confirm distinct anterior–posterior endoderm patterning

Microarray analysis was applied to compare HFOs to human embryonic hearts at 5–7 weeks of gestation (the earliest samples available owing to ethical and practical considerations); the 100 most significantly up/downregulated genes between HFOs, human hearts and hPSCs (control group) were assessed (Extended Data Fig. 4b). The majority of the 46 genes downregulated in HFOs compared to the heart samples represented cardiac markers, in particular those related to cardiovascular homeostasis, sarcomere proteins and cardiac ion channels, including *NPPA* (natriuretic peptide A), *SLC8A1* (sodium/calcium exchanger 1), *CLIC5* (chloride intracellular channel 5), *TTN* (titin), *MYH7* (MHCβ), *TNNT2* (cTnT) and others.

This observation, first, most likely reflects the progressed developmental stage of the hearts (comprising the fully folded, four-chambered organ) compared to HFOs. Second, the human samples represent micro-surgically isolated hearts lacking any other tissues, whereas HFOs contain endodermal components as outlined above. Thus, conversely, most of the 21 genes upregulated in HFOs represented endodermal genes, particularly those specific to liver, including *HNF1A* (hepatocyte nuclear factor 1α), *APOA1* (apolipoprotein A1) and *TF* (transferrin). The 21 genes that were similarly upregulated in both HFOs and heart samples compared to hPSCs included cardiac transcription factors such as *GATA4*, *GATA6* and *HAND2*; conversely, most of the 12 genes downregulated in HFOs and human hearts were pluripotency-related markers such as *POU5F1* and *LCK*.

**Fig. 4:**
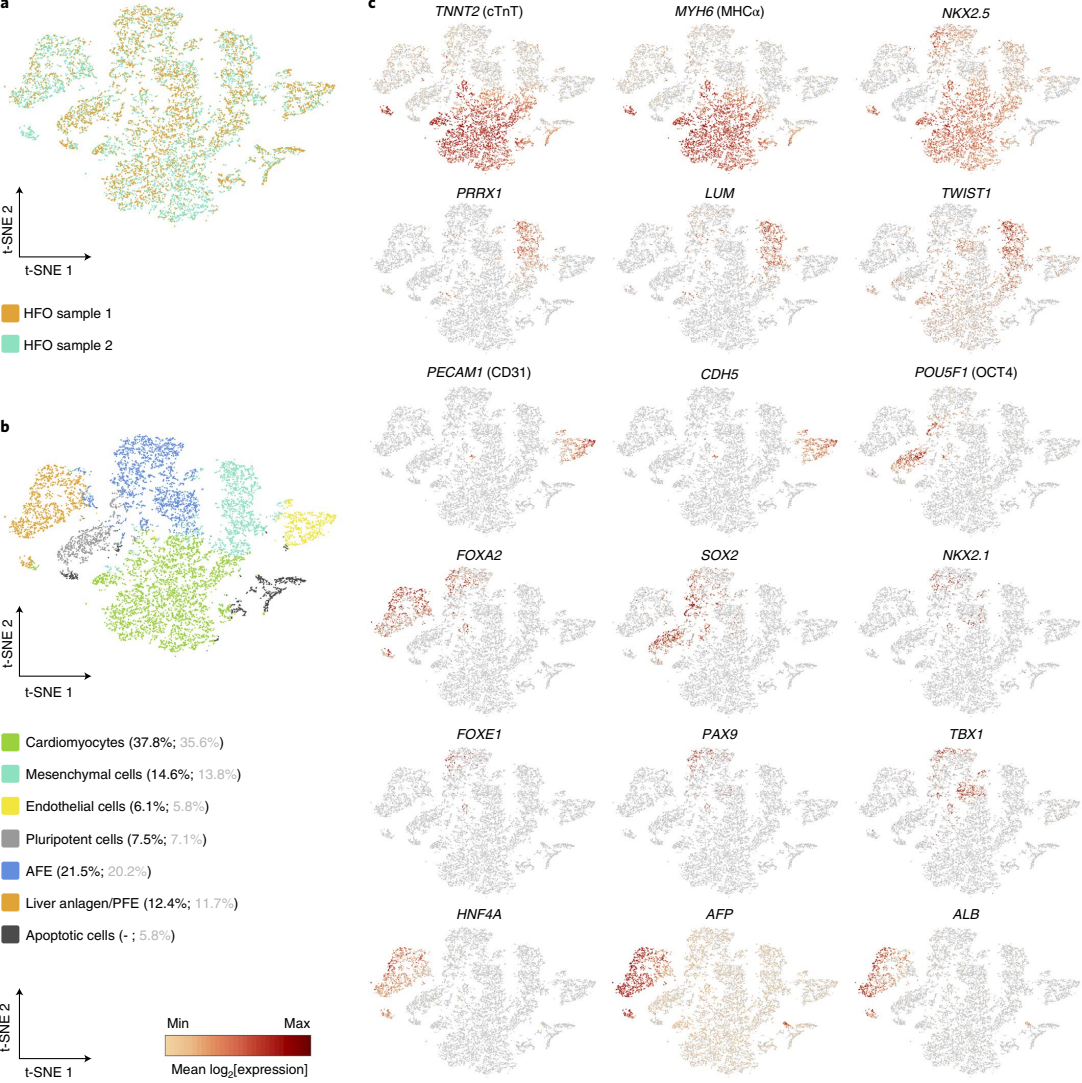
scRNA-seq confirms distinct anterior–posterior endoderm patterning. **a**, A t-SNE plot showing an overlay of two d13 HFO samples in different colors (HFO sample 1: 7,505 cells; HFO sample 2: 4,605 cells). **b**, A t-SNE plot of composite scRNA-seq data from both HFO samples colored by automated clustering. The naming of each cluster was based on the expression of key genes in these cell populations. The percentages of respective cell populations among all living cells (black) or all cells including apoptotic cells (gray) are listed in brackets behind each cluster. **c**, The relative expression of the indicated genes across all HFO clusters shown in the composite t-SNE plots. The dark red color equals a high level of expression of the indicated gene.

To explore the identity of tissues in HFOs in more detail, single-cell RNA sequencing (scRNA-seq) was applied on two HFOs. An overlay of the t-distributed stochastic neighbor embedding (t-SNE) plots of both samples showed equally positioned cell populations, corroborating the robustness of tissue formation in individual HFOs (Fig. 4a). Automated clustering revealed seven clusters (Fig. 4b), of which one showed significant overexpression of mitochondrial genes representing dead cells. The other six clusters showed population-specific expression patterns confirming the microarray analysis and IF staining results. t-SNE plots of key genes expressed by these clusters are presented in Fig. 4c and Extended Data Fig. 4c. The largest cluster (~38% of all living cells) was characterized by numerous cardiac genes including *TNNT2* (cTnT), *MYH6* (MHCα) and *NKX2.5*. *ISL1* expression in this cluster was low, which is consistent with the flow cytometry data of d13 HFOs shown in Extended Data Fig. 2b. Expression of *TBX5* and *HCN4* was also found in this cluster; these markers have been primarily associated with the FHF but were reported for the SHF as well^27,28^.

A separate cluster (~15% of all living cells) was specified by a pattern typical of mesenchymal, epithelial-to-mesenchymal transition and extracellular matrix markers including *PRRX1*, *LUM* (lumican) and *TWIST1*, whereas another cluster (~6%) was found to express endothelial-specific genes such as *PECAM1* and *CDH5*. scRNA-seq also revealed a cluster (~8%) specified by typical pluripotency markers such as *POU5F1* and *SOX2*, suggesting the maintenance of undifferentiated hPSCs in HFOs.

The remaining two clusters were characterized by the common expression of *FOXA2*, thus representing endodermal populations. The first endodermal cluster (~22% of all living cells) was found to express a variety of AFE-specific genes including *SOX2*, *NKX2.1* (lung progenitors), *FOXE1* (esophageal progenitors), *PAX9* (lung and esophageal progenitors; pharyngeal endoderm), *TBX1* (pharyngeal endoderm) and *ISL1*, which is expressed by pharyngeal endoderm in addition to the SHF. Equivalently to the dual expression of *NKX2.5* in cardiac progenitors and the pharyngeal endoderm in the embryo^29^, this marker was found to be expressed in both the cardiomyocyte and the AFE cluster in HFOs. However, although AFE-specific gene expression for markers such as *NKX2.1* and *NKX2.5* was revealed by scRNA-seq, expression of these markers in the IC was not detected on the protein level, neither by IF staining specific to NKX2.1 nor by the NKX2.5–eGFP reporter. This suggests that AFE progenitors found at the current developmental stage of HFOs represent an early maturation state. Focusing on *SOX2*, which also marks neuronal cells, we further analyzed our scRNA-seq data with respect to the neuronal markers *SOX1* and *PAX6*, but found only very few positive cells scattered across all clusters.

The second endodermal cluster revealed by scRNA-seq (~12% of all living cells) was characterized by the expression of numerous liver-specific genes, including *HNF4A*, *AFP* (alpha fetoprotein) and *ALB* (albumin). A small proportion of cells in this cluster were found to express *PDX1*, a marker of pancreas, which originates from the PFE in addition to liver anlagen^30^. Finally, almost no expression of the mid/hindgut marker *CDX2* was observed. Together, these results reinforce the presence of distinct AFE and PFE tissues in addition to the heart anlagen in HFOs.

**Extended Data Fig. 5:**
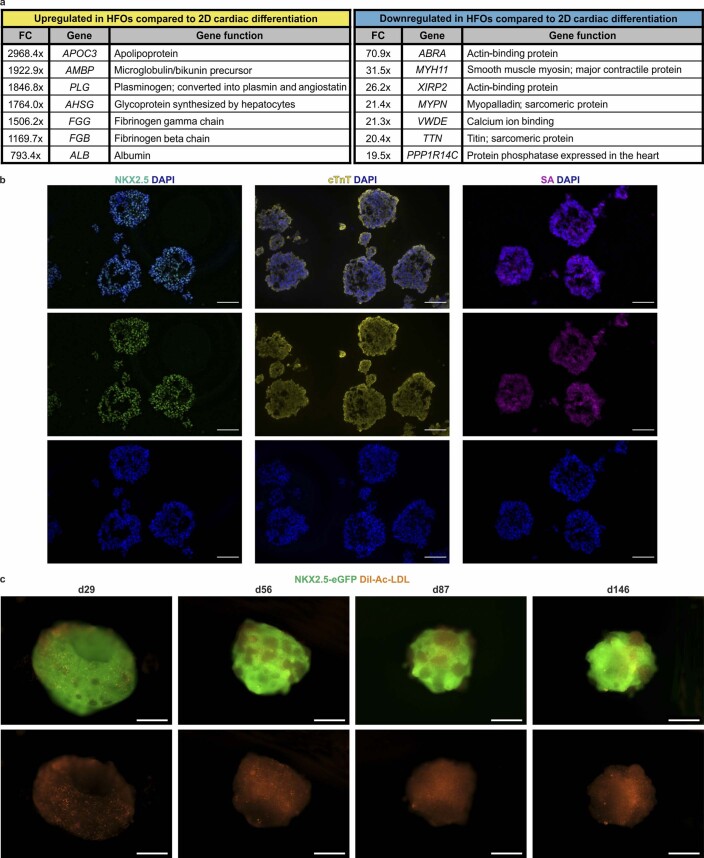
Comparison to conventional cardiac differentiation and long-term culture of HFOs. **a**, Selected genes, which were up- or downregulated in HFOs compared to cardiomyocytes generated by a conventional 2D differentiation protocol and respective gene functions according to the GeneCards database^38^. FC= fold change. **b**, Cryosections of aggregates generated by a conventional 3D cardiac differentiation protocol stained for the cardiac markers NKX2.5, cTnT and sarcomeric actinin (SA). **c**, Long-term culture of HFOs. HES3 NKX2.5-eGFP-derived HFOs were cultured up to 146 days in suspension after dissolving the surrounding Matrigel. HFOs were stained with DiI-Ac-LDL to visualize endothelial cells. Scale bars: **b**: 100 µm; **c**: 500 µm.

Applying microarray analysis, we then compared HFOs to conventional WNT modulation-directed 2D cardiac differentiation (resulting in >90% cardiomyocyte content^17^), revealing 572 differentially expressed genes (fold change ≥ 10; *q* ≤ 0.01). Similarly to the comparison of HFOs to human embryonic hearts (Extended Data Fig. 4b), genes encoding cardiac markers such as sarcomere proteins were downregulated in HFOs, whereas endoderm markers were upregulated (Extended Data Fig. 5a). Non-matrix-embedded cardiac differentiation of hPSCs in 3D suspension culture^16^ resulted, as expected, in almost pure cardiomyocytes as shown by IF staining for NKX2.5, cTnT and sarcomeric actinin (Extended Data Fig. 5b). Together, these results contrast the difference between the established conventional cardiac differentiation protocols and our strategy, enabling the formation of complex, highly structured HFOs.

To monitor how the morphology and tissue composition change over time, HFOs were kept in culture up to 146 days (Extended Data Fig. 5c). For practical reasons, the Matrigel surrounding the HFOs was dissolved (otherwise, it disrupts over time) and the HFOs were transferred to suspension culture plates; to visualize ECs, the vital dye DiI-acetylated low-density lipoprotein (DiI-Ac-LDL) was added. During the long-term culture period, HFOs survived and continued beating, and ECs were still present throughout the HFOs. However, the HFOs shrank in dimensions and changed in shape. Moreover, NKX2.5–eGFP-positive cells became dominant whereas NKX2.5–eGFP-negative cells progressively disappeared. This suggests that cardiomyocytes overgrew the endodermal components over time, which is probably a consequence of the applied culture medium previously optimized for cardiomyocyte support^16,31^. Together, these findings indicate that the applied culture conditions are not appropriate to maintain the early structure and tissue composition of HFOs, suggesting the requirement of future adaptions to support survival and eventually maturation of all tissue anlagen currently identified.

### *NKX2.5*-KO HFOs recapitulate aspects of the respective phenotype in mice

We tested the applicability of HFOs to model aspects of an *NKX2.5*-KO phenotype in vitro. In the embryo, NKX2.5, in interplay with other transcription factors, controls the expression of cardiac structural genes, including those encoding sarcomeric actinin, MHC and troponins. A constitutive *Nkx2.5* KO in mice leads to early embryonic lethality due to morphological defects, including failure of heart tube looping^32^. In contrast, mice with a conditional *Nkx2.5* KO (*Nkx2.5* deletion only in ventricular cells) survive but show conduction and morphological defects, including trabecular overgrowth and hypertrophy^33^.

**Fig. 5:**
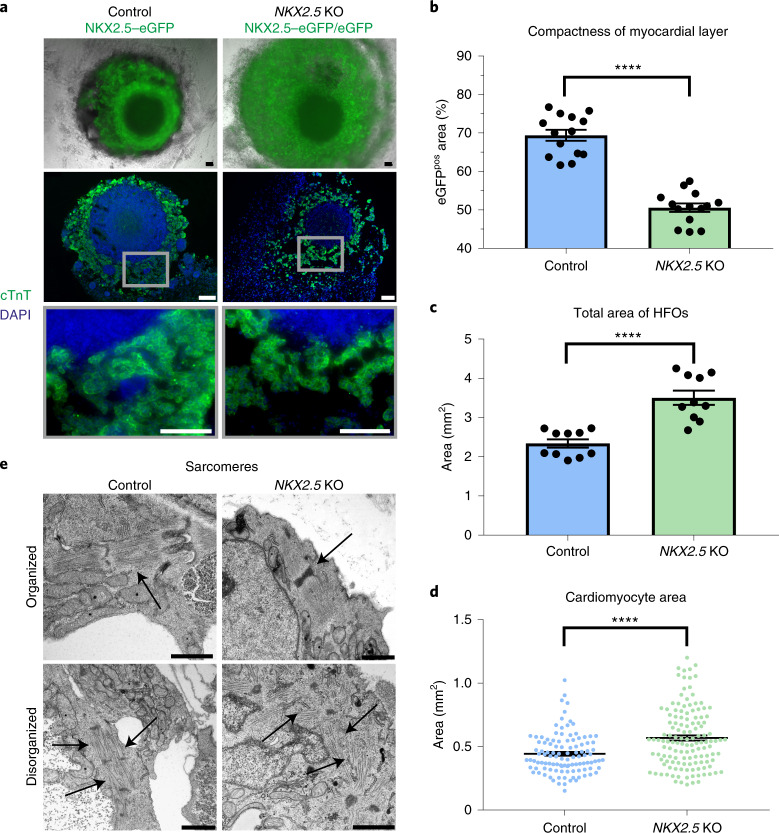
*NKX2.5*-KO HFOs recapitulate aspects of the respective phenotype in mice. **a**, *NKX2.5*-KO organoids appear less compact than control HFOs as shown on whole HFOs (first row) and on paraffin sections stained for cTnT (second and third rows). **b**–**d**, A comparison of ML compactness (**b**), total area (**c**) and cardiomyocyte (eGFP-positive cell) area (**d**) between control and *NKX2.5*-KO organoids on d10 of differentiation applying a two-tailed *t*-test. **b**, Control: *n* = 14 HFOs, *NKX2.5* KO: *n* = 14 HFOs from 3 independent experiments; **c**, control: *n* = 10 HFOs, *NKX2.5* KO: *n* = 10 HFOs from 2 independent experiments; **d**, control: *n* = 109 cells from 3 HFOs, *NKX2.5* KO: *n* = 133 cells from 4 HFOs derived from 1 experiment; the data are presented as mean ± s.e.m.; ****P ≤ 0.0001. **e**, TEM images of sarcomeres (arrows) in control and *NKX2.5*-KO organoids. Scale bars, 100 µm (**a**) and 1 µm (**e**).

**Extended Data Fig. 6:**
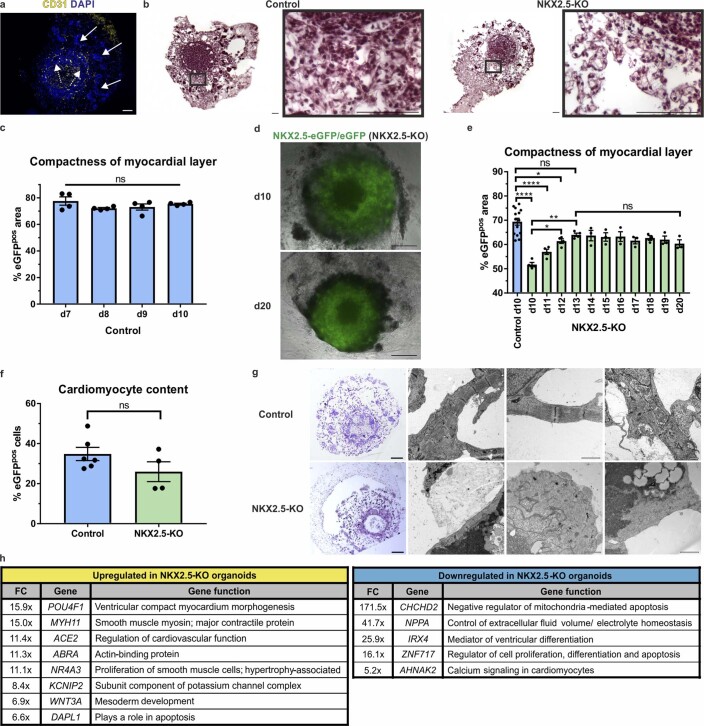
NKX2.5-KO HFOs recapitulate aspects of the respective phenotype in mice. **a**, Paraffin section of an NKX2.5-KO organoid stained for CD31 to visualize the vascular structures. Dotted arrows point at endodermal cavities in the inner core, solid arrows point at endodermal islands in the outer layer. **b**, Hematoxylin-eosin staining of HFOs and enlargements highlighting the reduced myocardial layer (ML) compactness in NKX2.5-KO organoids compared to control HFOs. **c**, ML compactness of control HFOs from d7 – d10 of differentiation. n = 4 HFOs from one experiment; one-way ANOVA (Tukey’s multiple comparison test); data are presented as mean ± SEM; ns = not significant (P>0.05). **d**, Exemplary images of an NKX2.5-KO organoid on d10 and d20 of differentiation. **e**, ML compactness of NKX2.5-KO organoids over time compared to the ML compactness of d10 HFOs. Control: n = 14 HFOs, NKX2.5-KO: n = 3 (d10 – d13) or 4 (d14 – d20) HFOs from 3 (control) or 1 (NKX2.5-KO) experiments; one-way ANOVA (Tukey’s multiple comparison test); data are presented as mean ± SEM; Control d10/ KO d12: *P = 0.0196; KO d10/d12: *P = 0.0269; KO d10/d13: **P = 0.0016; ****P ≤ 0.0001; ns = P > 0.05. **f**, Cardiomyocyte (eGFP-positive cell) content in NKX2.5-KO versus control HFOs determined by flow cytometry. Control: n = 6 HFOs, NKX2.5-KO: n = 4 HFOs from 2 independent experiments; two-tailed t-test; data are presented as mean ± SEM; ns = not significant (P > 0.05). **g**, Light microscopy (LM; overview in left column; toluidine blue staining) and transmission electron microscopy (TEM) pictures of sarcomeres in control and NKX2.5-KO organoids. **h**, Microarray analysis of control versus NKX2.5-KO organoids: Selected genes, which were up- or downregulated in NKX2.5-KO organoids and respective gene functions according to the GeneCards database^38^. FC=fold change. Scale bars: **a**, **b**: 100 µm; **d** = 500 µm; **g** = 200 µm (LM) or 1 µm (TEM).

**Extended Data Fig. 7:**
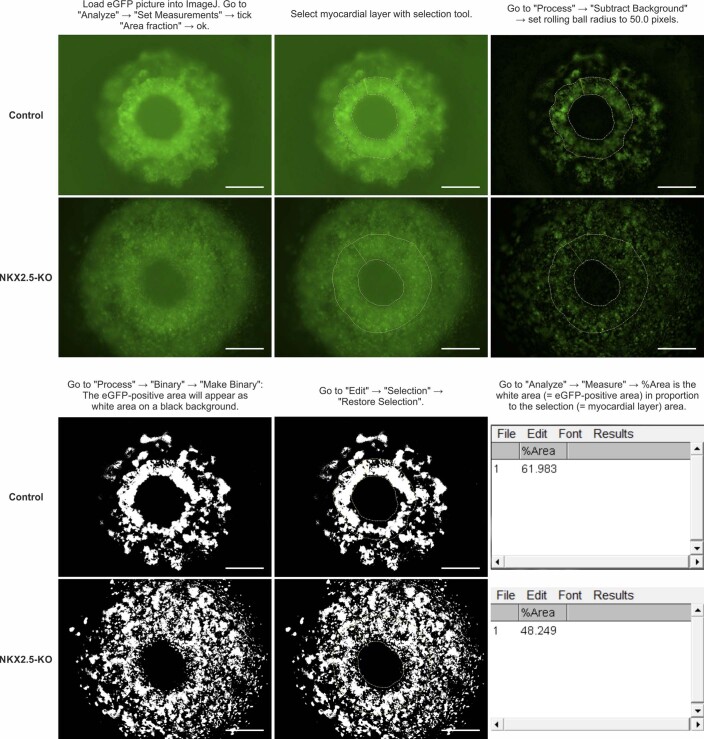
Defining the myocardial layer compactness of control versus NKX2.5-KO HFOs. The myocardial layer (ML) compactness was defined as the eGFP-positive area in proportion to the ML area. The figure shows stepwise how the compactness was determined using ImageJ software. Scale bars: 500 µm.

When generating HFOs from an *NKX2.5*-KO hESC reporter line (both alleles disrupted by *eGFP* insertion)^34^, we noted the formation of a layered pattern equivalent to heterozygous NKX2.5–eGFP controls (Fig. 5a), including a vascular network in the IC and the formation of spatially distinct endodermal tissues (Extended Data Fig. 6a). However, *NKX2.5*-KO organoids appeared less compact (Fig. 5a). Particularly in the ML, the adhesion between cardiomyocytes seemed to be decreased, as indicated by the analysis of whole HFOs and paraffin sections stained for cTnT/4’,6-diamidino-2-phenylindole (DAPI; Fig. 5a) or hematoxylin–eosin (H&E; Extended Data Fig. 6b). ImageJ-based analysis of the ML structure confirmed the significant loss of tissue compactness in response to *NKX2.5* disruption (Fig. 5b and Extended Data Fig. 7), which is reminiscent of findings in *Nkx2.5*-KO mice showing a decreased cell adhesion within the myocardium before heart tube looping^32^.

Assessing the general development of tissue compactness in the ML of control HFOs in a time-resolved manner revealed no measurable changes from d7 (upcoming NKX2.5–eGFP expression) to d10 (Extended Data Fig. 6c). However, analyzing *NKX2.5*-KO organoids from d10 to d20 by the same assay revealed a significant increase of compactness over time reaching a maximum on d13 (matching the compactness of d10 controls; Extended Data Fig. 6d,e) and remaining in a steady state thereafter.

When quantifying the total area of HFOs in the front view, we noted that *NKX2.5*-KO organoids were overall significantly larger compared to controls (Fig. 5c). We did not observe an increased cardiomyocyte content in *NKX2.5*-KO organoids (Extended Data Fig. 6f), suggesting that their increase in size was not due to an increase in cardiomyocyte induction and/or proliferation but was a result of the decreased tissue compactness. As conditional *Nkx2.5* loss in mice has been reported to induce hypertrophy in cardiomyocytes on the cellular level^35^, we analyzed whether this pertains to our model as well: determining the cell surface area of HFO-derived plated cardiomyocytes indeed revealed significant enlargement of *NKX2.5*-KO-derived cells compared to controls (Fig. 5d).

Together, these findings suggest that our model resembles aspects of the decreased cardiomyocyte adhesion and hypertrophy resulting from NKX2.5 loss in native heart development.

TEM further showed that both *NKX2.5*-KO and control organoids contained organized as well as disorganized sarcomeres in cardiomyocytes (Fig. 5e and Extended Data Fig. 6g). However, while organized sarcomeres predominated in controls, these structures appeared substantially more disarrayed in *NKX2.5*-KO organoids. Still, beating patterns similar to those of controls were observed in the KO approach (Supplementary Video 6), suggesting that NKX2.5 per se is not essential for contractility.

Microarray analysis of control versus *NKX2.5*-KO organoids identified 391 differentially expressed genes (fold change ≥ 2; *P* ≤ 0.05). As expected, the well-known NKX2.5 target genes *NPPA* and *IRX4* were highly downregulated in *NKX2.5*-KO organoids (41.7-fold or 25.9-fold, respectively). Anderson et al.^34^ found that NKX2.5 favors a ventricular-like cardiac phenotype and represses smooth muscle cell differentiation. In agreement, we observed a 15.0-fold upregulation of the smooth muscle myosin gene *MYH11* (myosin heavy chain 11). In addition, we detected an 11.1-fold upregulation of *NR4A3*, a nuclear hormone receptor promoting smooth muscle cell proliferation^36^, in our KO model. *NR4A3* upregulation has been associated with cardiac hypertrophy^37^, suggesting that this might also relate to the enlargement of cardiomyocytes in *NKX2.5*-KO organoids. Whereas the expression of genes encoding structural proteins such as myosin light chain (MLC) 1A, MLC1V, MLC2A, cardiac muscle alpha actin and MHCα and β showed comparable levels between control and *NKX2.5*-KO organoids, expression of MLC2V (encoded by *MYL2*) was slightly decreased in the KO approach (1.6-fold), reflecting observations in different mouse models^32,33^. Further details on differentially expressed genes and their respective functions (according to the GeneCards database^38^) are presented in Extended Data Fig. 6h.

## Discussion

**Fig. 6:**
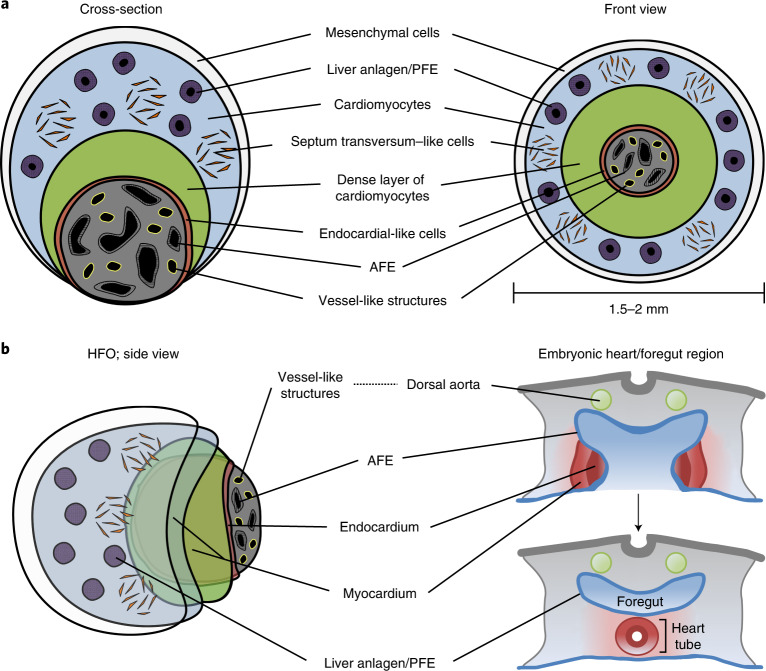
HFOs resemble early heart and foregut anlagen. **a**, A scheme of a typical HFO shown as a cross-section and in front view. **b**, A schematic comparison of an HFO with the early embryonic heart/foregut region (transverse plane).

**Extended Data Fig. 8:**
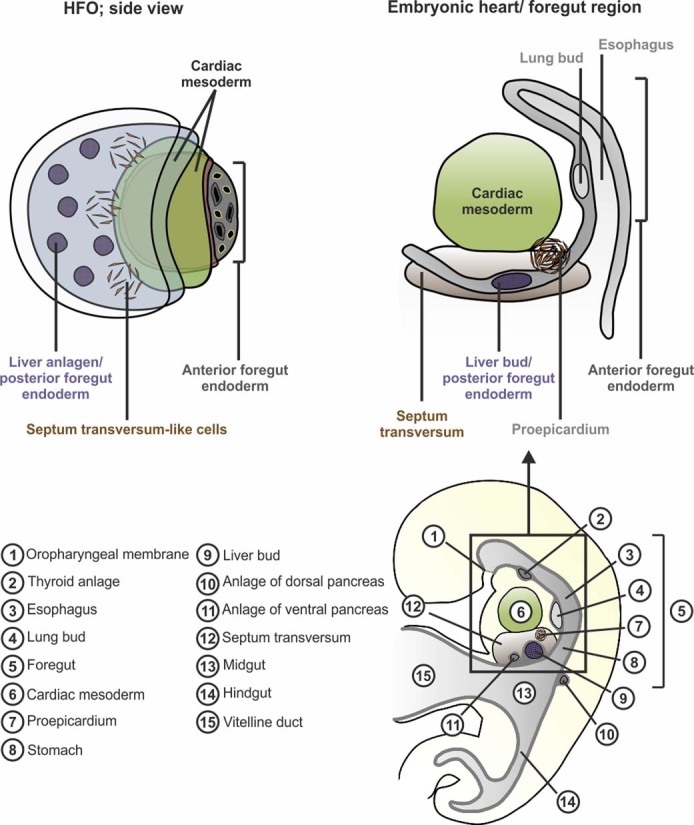
HFOs resemble early heart and foregut anlagen. Schematic comparison of an HFO with the early embryonic heart/ foregut region (sagittal plane).

We here describe a robust method to induce self-organizing hPSC-derived HFOs, which are composed of an ML lined with endocardial-like cells, surrounded by ST-like cells, framed by distinct AFE and PFE tissues, and pervaded by a vascular network (Fig. 6a). The HFOs show morphological similarities to early heart and foregut anlagen in the developing embryo (Fig. 6b and Extended Data Fig. 8) where, after precardiac mesoderm specification, cardiac progenitors form the heart-forming regions in close proximity to the developing AFE^13^. Signals from the AFE mediate the delamination of endocardial precursor cells from cardiac mesoderm, such that endocardial cells become localized between the developing myocardium and the AFE^39^. Similarly, in HFOs, endocardial-like cells form a lining between the ML and the IC (comprising AFE tissue), although further assessment will be necessary to unequivocally prove that these cells represent endocardium.

Later in development, endodermal folding leads to the formation of the foregut pocket. Meanwhile, cells of the heart-forming regions fuse into the cardiac crescent and subsequently form the heart tube, which consists of a myocardial and an endocardial layer^13^.

While signals from the foregut endoderm are required for proper myo- and endocardial patterning, signals emitted from the cardiac mesoderm are in turn necessary to induce lung anlagen in the AFE and liver anlagen in the PFE^40^. Reminiscent of this signal interchange, HFOs show a distinct anterior–posterior endoderm patterning, with AFE anlagen being located in the IC, whereas liver progenitors form rosettes in the OL. In addition to these liver progenitors, we found WT1-expressing cells in the OL, which seems to resemble embryonic patterns in that WT1 is expressed by a variety of embryonic tissues (that is, the ST and its derivatives) forming in close proximity to (and involved in the development of) the liver and heart anlagen. For example, induction of the liver bud requires—apart from cardiac mesoderm-derived growth factors—signals emitted from the ST^41^. In turn, signals derived from the liver bud induce formation of the proepicardium, which emerges as a cluster of cells from the ST mesenchyme in close proximity to the heart tube^14,42^. WT1 is expressed not only by the ST and its derivatives but also by the intermediate mesoderm and the developing kidney^43^. Formation of the ST lineage seems most likely in HFOs (owing to the proximity to liver and myocardium anlagen), but the exact identity of WT1-expressing cells in our model remains to be confirmed.

Vascular network formation is another hallmark of HFOs. In the embryo, the first recognizable vessels are the bilaterally paired dorsal aortae developing in close proximity to the foregut endoderm^13^. The aortae-forming ECs are derived from splanchnic mesoderm equivalent to heart precursors. Vasculogenesis of these cells is stimulated by factors originating from the underlying endoderm and the lateral plate mesoderm^44^. Accordingly, the interplay of foregut endoderm found in the IC of HFOs and the lateral plate mesoderm derivatives (that is, the ML) might provide the instructive signals directing the formation of VLs found predominantly within the IC.

Apparent differences between HFOs and native heart/foregut anlagen in the embryo include morphological and temporal aspects. For example, on the one hand, the morphology of the ML lined with endocardial-like cells in HFOs is reminiscent of a pre-heart-tube-like stage at ~2–3 weeks of human gestation^14^ resembling a single heart-forming region. On the other hand, in the human embryo, liver bud development is not initiated before 3–4 weeks of gestation, which is after heart tube formation^26^. Furthermore, HFOs do not comprise a single foregut tube but rather two spatially independent foregut endoderm compartments (that is, AFE in the IC versus liver progenitors/PFE in the OL). Thus, no proper anterior–posterior axis is formed in HFOs; the heart anlage (that is, the ML) is intercalated between the endoderm compartments rather than being located ventrally to the foregut tube as in the embryo. A further limitation of the HFO model is the incomplete patterning of the heart anlagen. While the ML is formed by SHF-like cells, the presence of FHF-like cells seems missing, which is probably a consequence of our WNT modulation-based differentiation protocol^45^. However, in the embryo, the FHF forms adjacent to the AFE, whereas SHF cells appear medially to the FHF^46^. Last, formation of cardiac jelly, which develops around week 4 of human gestation between the myocardium and endocardium^47^, was not apparent in HFOs, potentially reflecting the early developmental stage. Despite these differences from spatiotemporal heart and foregut development in vivo, our HFO approach mimics important aspects of native pattern formation.

Further development and maturation of HFOs will require modification of the differentiation protocol, including additional, specific (bio)chemical and mechanical cues. Challenges for advancing the protocol include overcoming the degeneration of HFOs over time and achieving directed progression to heart tube formation. In the embryo, the heart tube develops by fusion of the bilateral heart-forming regions. Each heart-forming region is capable of developing into a complete four-chambered heart without midline fusion^48^, suggesting that our model has the potential to progress to the next stages of heart development.

Tissue and organ development are dependent on (bio)chemical and mechanical cues that regulate proper spatiotemporal patterning. Minor protocol modulations, such as changes of the bulk cell density only during the 24 h of CHIR-induced hPSC differentiation, can shift primitive-streak-like priming into either more endoderm-like or more paraxial mesoderm-like cell fates at the expense of cardiac mesoderm formation^17^. These lineage variations seem to be mediated by cell-secreted paracrine factors downstream of the CHIR-based WNT pathway activation, indicating the broad developmental potential that can result from such a simple differentiation strategy^17,49^. Successful HFO formation depends on Matrigel encapsulation combined with the exact interplay of several defined cues, such as the aggregate size (based on the number of hPSCs seeded per well on d–4), culture medium titration (resulting in a specific bulk cell density) and process timing. HFO differentiation failed on embedding in Geltrex, a growth-factor-reduced, Matrigel-like ECM preparation. This indicates that proper HFO patterning is dependent on one or more unidentified factors in Matrigel. Future identification of these signals would enable fully defined conditions for HFO formation and help elucidate additional mechanisms involved.

We presented an application of HFOs to study genetic defects in vitro by demonstrating that *NKX2.5*-KO HFOs show a phenotype reminiscent of cardiac malformations observed in transgenic mice, including decreased cardiomyocyte adhesion and hypertrophy. Such findings were not revealed by the recent characterization of the same HES3 *NKX2.5*-KO reporter line when differentiated into cardiomyocytes under conventional conditions^34^. Contractility seemed not impaired in *NKX2.5*-KO HFOs, which is consistent with findings by Anderson et al. in the hESC model^34^ and by studies in mice as well^32^. It is worth noting, however, that mutations in *NKX2.5* have also been associated with contraction defects; for example, Briggs et al.^50^ have reported that perinatal loss of *Nkx2.5* resulted in contraction defects in mice, which cannot be modeled with the current stage of our early developmental model. We furthermore observed an upregulation of *NR4A3* in *NKX2.5*-KO organoids. In contrast to our results, upregulation of *NR4A3* on *NKX2.5* disruption was not observed by the in vitro approach by Anderson et al.^34^ and, to our best knowledge, has not been reported by previous in vivo studies. Together, our observations highlight the utility of HFOs for the in vitro modeling of gene KO phenotypes. However, it should be noted that the modeling capabilities of HFOs are currently limited to very early developmental stages. HFO generation is relatively simple, fast, robust and applicable to different independent hPSC lines. Our protocol seems amenable to automatization and medium- to high-throughput screenings in the future, opening new perspectives for investigating mechanisms of human embryogenesis, disease modeling and drug discovery.

## Methods

### Formation and culture of HFOs

Experiments using hESC lines were performed under allowance ‘108: Genehmigung nach dem Stammzellgesetz’ granted by the Robert Koch Institute. HES3 NKX2.5–eGFP^19,51^, HSC_ADCF_SeV-iPS2^20^, HES3 MIXL1–GFP^18^ and HES3 NKX2.5–eGFP/eGFP^34^ (*NKX2.5*-KO) cells were maintained on irradiated mouse embryonic fibroblasts. Before formation of HFOs, cells were transferred to Geltrex-coated flasks in Essential 8 (E8) medium^52^ (DMEM/F-12, HEPES with 64 mg l^−1^ ascorbic acid-2-phosphate, 100 μg l^−1^ bFGF, 20 mg l^−1^ insulin, 543 mg l^−1^ NaHCO_3_, 14 μg l^−1^ Na_2_SeO_3_, 10.7 mg l^−1^ transferrin, 2 μg l^−1^ TGFβ) supplemented with 10 µM Y-27632 (Tocris). Cells were passaged twice per week and the medium was changed daily. For HFO formation, on d−4, cells were detached from Geltrex-coated flasks using Accutase (Thermo Fisher Scientific). A total of 5,000 cells per well were seeded in a U-shaped ultralow-attachment 96-well plate (Thermo Fisher Scientific) in E8 medium supplemented with 10 µM Y-27632. The 96U-well plate was centrifuged at 300*g* and 4 °C for 3 min and placed in the incubator to let one aggregate per well form overnight. On d−2, each aggregate was embedded in a Matrigel droplet (Corning; catalog number: 354234). The following Matrigel lots were applied: no. 8176013, no. 8113018, no. 7002568, no. 6116005 and no. 5131445. After embedding, the plate was placed in the incubator for 1 h to let the Matrigel solidify. Afterwards, E8 medium was added to the embedded aggregates. Differentiation of these aggregates was initiated on d0 by replacing the medium with RPMI/1640 medium (Thermo Fisher Scientific) containing 2% B-27 supplement without insulin (RB−) (Thermo Fisher Scientific) and supplemented with 7.5 µM CHIR (synthesized by the Institute for Organic Chemistry, Leibniz University Hannover). After 24 h, the medium was exchanged by RB−. On d3, RB− supplemented with 5 µM IWP2 (Tocris) was added for 48 h and exchanged by RB− on d5. From d7 onwards, aggregates were cultivated in RPMI/1640 medium containing 2% B-27 supplement (RB+) (Thermo Fisher Scientific). Differentiation was completed on d10. HFOs were analyzed between d10 and d13. Pictures of whole HFOs were taken with an Axio Observer A1 (Zeiss) or at an Olympus CKX41 inverted microscope (Olympus).

### Calcium imaging of HFOs

For calcium imaging, the Rhod-4 Calcium Assay Kit (Abcam) was used. Rhod-4 dye-loading solution was prepared according to the manufacturer’s instructions. HFOs were incubated in Rhod-4 dye-loading solution for 1 h in the incubator. Afterwards, the Rhod-4 dye-loading solution was exchanged by RB+. After another 1 h in the incubator, HFOs were analyzed. Videos of beating HFOs were taken with an Observer Z1 microscope (Zeiss).

### Long-term culture and DiI-Ac-LDL staining of HFOs

On d10 of differentiation, HFOs were transferred to an ultralow-attachment 24-well plate (Thermo Fisher Scientific) and washed with 1× phosphate-buffered saline without Mg^2+^/Ca^2+^ (PBS w/o), and the Matrigel surrounding the HFOs was dissolved using the Cell Recovery Solution (Corning) according to the manufacturer’s instructions. Afterwards, HFOs were washed again with PBS w/o and cultured in RB+ for up to 146 days. Medium changes were performed twice per week. To mark and visualize ECs, HFOs were stained with 5 µg ml^−1^ DiI-Ac-LDL (Thermo Fisher Scientific) in RB+ for 4 h in the incubator. Afterwards, HFOs were washed with PBS w/o and analyzed at the Axio Observer A1.

### Culturing HFOs in different 3D matrices

Apart from Matrigel, HFOs were embedded in Geltrex (15 mg ml^−1^ protein concentration; Thermo Fisher Scientific) or in human collagen I (3 mg ml^−1^ protein concentration; Sigma-Aldrich) on d−2 of differentiation. For embedding in Geltrex, an equivalent protocol was applied as for Matrigel. The human collagen I solution was neutralized with NaOH before embedding (50 µl collagen was mixed with 10.8 µl 0.1 M NaOH); after embedding, the culture plate was placed for 30 min in the incubator to let the collagen solidify.

### Whole-mount IF staining and clearing

HFOs were fixed with 4% paraformaldehyde (PFA) overnight at 4 °C, washed with Tris-buffered saline (TBS) and incubated with blocking buffer (TBS containing 5% BSA and 0.25% Triton X-100) overnight at 4 °C. Afterwards, incubation with primary antibodies was performed in staining buffer (TBS containing 1% BSA) for four days at 4 °C. The primary antibody solution was renewed after two days of incubation. Primary antibodies were used at the following dilutions: anti-CD31 (Agilent GA610, 1:20), anti-cTnT (Thermo Fisher Scientific MA5-12960, 1:100), anti-(pan) myosin heavy chain (Hybridoma Bank MF20, 1:10), anti-NKX2.5 (Cell Signaling Technology 8792, 1:800), anti-vimentin (abcam ab92547, 1:500). The HFOs were then washed two times for 1 h each with TBS at 4 °C and incubated with the secondary antibodies in staining buffer containing 0.57 µg ml^−1^ DAPI for two days at 4 °C. Secondary antibodies were donkey Alexa Fluor 488 (Jackson ImmunoResearch, 1:500) and donkey Cy3 (Jackson ImmunoResearch, 1:300) conjugates. Thereafter, HFOs were washed three times for 1 h each with TBS at 4 °C. Images were either directly taken at an Axio Observer A1 (Zeiss) or samples were stored at 4 °C for further processing. For imaging via multiphoton microscopy (MPM) and SLOT, tissue clearing of HFOs was performed. Therefore, the HFOs were dehydrated by applying ethanol diluted in water in increasing concentrations (25%, 50% and 75%) for 1 h each at room temperature and kept in 99% ethanol overnight at room temperature. The next day, dehydrated HFOs were incubated in a series of increasing concentrations of MSBB (50% methyl salicylate/50% benzyl benzoate) in ethanol (25%, 50% and 75%) for 1 h each at room temperature and kept in 100% MSBB overnight at room temperature. Cleared HFOs were stored in 100% MSBB at 4 °C until imaging with MPM or SLOT. For MPM via TriM Scope II (LaVision Biotec), two-photon fluorescence was applied using an excitation wavelength of 780 nm and a 460/50 band-pass filter for DAPI and a 565/24 band-pass filter for Cy3. For SLOT^53^, the excitation wavelength was 450 nm and a 525/26 band-pass filter was applied.

### Staining of paraffin sections

HFOs and E11.5 mouse embryos were fixed with 4% PFA overnight at 4 °C, washed with TBS and embedded in paraffin. Tissue sections of 3 µm in thickness were generated using the Leica RM2245 microtome (Leica).

Tissue sections were stained with H&E using standard protocols or used for IF staining. For the latter, antigen retrieval was performed in pH 9 citrate buffer (10 mM sodium citrate tribasic dehydrate and 1 mM EDTA) at 96 °C for 20 min. Afterwards, tissue sections were incubated with blocking buffer for 1 h at room temperature followed by overnight incubation with primary antibodies in staining buffer at 4 °C. Primary antibodies were used at the following dilutions: anti-cTnT (Thermo Fisher Scientific MA5-12960, 1:100), anti-GFP (OriGene R1091P, 1:250), anti-WT1 (Santa Cruz sc-393498, 1:50) and anti-SOX17 (R&D systems AF1924, 1:200). Staining with secondary antibodies diluted in staining buffer was performed for 1 h at room temperature. Secondary antibodies were donkey Alexa Fluor 488 (Jackson ImmunoResearch, 1:300) and donkey Cy3 (Jackson ImmunoResearch, 1:200) conjugates. Then, tissue sections were counterstained with 1.7 µg ml^−1^ DAPI for 15 min at room temperature, mounted with mounting medium (Dako) and stored at room temperature overnight before analysis.

For NFATc1 staining, antigen retrieval was performed using pH 6 citrate buffer and the primary antibody solution (anti-NFAT2; abcam ab25916, 1:100) was renewed after overnight incubation at 4 °C. Tissue sections were incubated with the renewed antibody solution for 3 h at room temperature and again overnight at 4 °C.

For anti-CD31 staining (Agilent GA610, 1:20), antigen retrieval was performed with pH 9 citrate buffer followed by incubation with 3% H_2_O_2_ in TBS for 1 h at room temperature. As the secondary antibody, a HRP-labeled anti-mouse IgG antibody was used (Perkin Elmer NEF822001EA, 1:500) followed by signal development using TSA Cy3 (Perkin Elmer NEL760001KT, 1:50). Pictures were taken with the Axio Observer A1 (Zeiss) or at the Olympus FV1000 confocal microscope (Olympus).

Note that the pictures have been processed using AxioVision SE64 Rel. 4.8, Adobe Photoshop CC and ImageJ software to remove staining artifacts created by paraffin remnants.

### Cryosectioning and staining

One day before cryoembedding, the culture medium of HFOs was exchanged by RB+ containing 0.5 mg ml^−1^ dextran (Sigma-Aldrich). The next day, HFOs were washed with PBS w/o, incubated in Tissue-Tek (Sakura Finetek) for a few minutes, transferred to a cryomold filled with fresh Tissue-Tek, and frozen in a Microm HM 560 cryotome (Thermo Fisher Scientific). Frozen HFOs were stored at −80 °C until use. Tissue sections of 10 µm in thickness were generated using the Microm HM 560 cryotome, dried at room temperature overnight and stored at −80 °C until use.

For IF staining, cryosections were fixed with 4% PFA for 5 min at room temperature followed by incubation with blocking buffer for 1 h at room temperature and overnight incubation with primary antibodies in staining buffer at 4 °C. Primary antibodies were used at the following dilutions: anti-CD31 (Agilent GA610, 1:20), anti-SOX2 (Cell Signaling Technology 3579, 1:200), anti-HNF4α (abcam ab94748, 1:100), anti-cTnT (abcam ab64623, 1:200), anti-cTnT (Thermo Fisher Scientific MA5-12960, 1:100), anti-NKX2.5 (Cell Signaling Technology 8792, 1:800) and anti-α-actinin (sarcomeric; Sigma-Aldrich, A7811, 1:800). Staining with secondary antibodies diluted in staining buffer was performed for 1 h at room temperature. Secondary antibodies were donkey Alexa Fluor 488 (Jackson ImmunoResearch, 1:500) and donkey Cy3 (Jackson ImmunoResearch, 1:200) conjugates. Sections were counterstained with 1.7 µg ml^−1^ DAPI for 15 min at room temperature, mounted with mounting medium (Dako) and kept at room temperature overnight before analysis or storage at 4 °C. Pictures were taken with the Axio Observer A1 (Zeiss) and processed using AxioVision SE64 Rel. 4.8.

### Flow cytometry

HFOs were individually dissociated into single cells using the STEMdiff Cardiomyocyte Dissociation Kit (Stemcell Technologies) and stained by applying the Fix&Perm Kit (Nordic MUbio). Cells were measured at a BD Accuri C6 flow cytometer or at a MACSQuant Analyzer 10 (Miltenyi Biotec), followed by data analysis with FlowJo V10. The following primary or directly labeled antibodies were used: anti-CD31–APC (Miltenyi Biotec 130-092-652, 1:20), anti-ISL1/2 (DSHB 39.4D5, 1:250), anti-TBX5 (Abnova H00006910-M01, 1:100). Secondary antibodies were donkey Cy5 (Jackson ImmunoResearch, 1:200) conjugates.

### Electron microscopy and 3D reconstruction

Samples were fixed for 30 min at room temperature and overnight at 4 °C in 150 mM HEPES buffer (pH 7.35) containing 1.5% glutaraldehyde and 1.5% formaldehyde. After post-fixation in 1% osmium tetroxide for 2 h at room temperature and in 4% uranyl acetate at 4 °C overnight, samples were dehydrated in acetone and embedded in EPON. Sectioning was performed at a UC7 microtome (Leica). For 3D reconstruction, 2-µm-thick sections were collected on glass slides, stained with toluidine blue and imaged with a Zeiss Axio microscope (Zeiss) in the bright-field mode. Image alignment of consecutive sections was performed using ImageJ software. Serial section reconstruction was carried out using Reconstruct software^54^. The video was achieved from the 3D-Scene in Reconstruct using Camstudio software. For TEM, sections of 50 nm in thickness were collected on formvar-coated copper single-slot grids, post-stained with 2% uranyl acetate and lead citrate^55^ and observed in a Morgagni transmission electron microscope (FEI). Images were recorded at 80 kV using a 2,048 pixels × 2,048 pixels side-mounted Veleta charge-coupled device (CCD) camera, binned to 1,024 pixels × 1,024 pixels. For Fig. 2c–I, images of several parts of the VL were recorded separately and aligned to one picture using the Auto-Align tool in Adobe Photoshop CC. Scale bars of these images were removed afterward using the Clone Stamp Tool.

### Patch clamp analysis of HFO-derived cardiomyocytes

Action potentials of HFO-derived cardiomyocytes were recorded by the patch clamp technique in the whole-cell current clamp mode at room temperature. Therefore, HFOs were individually dissociated into single cells using the STEMdiff Cardiomyocyte Dissociation Kit (Stemcell Technologies) and seeded onto Geltrex-coated glass coverslips in a 12-well plate at a density of 20,000 cells per well in RB+ containing 10 µM Y-27632. Cells were analyzed 1–3 days after seeding using standard Tyrode’s solution in the recording chamber containing: 140 mM NaCl, 5.4 mM KCl, 1.8 mM CaCl_2_, 1 mM MgCl_2_, 10 mM HEPES, 10 mM glucose, adjusted with NaOH to pH 7.4. The intracellular solution contained: 120 mM K gluconate, 10 mM Na gluconate, 1 mM MgCl_2_, 10 mM HEPES, 10 mM EGTA/KOH, 3 mM Mg-ATP, with pH adjusted to 7.2 by KOH. Borosilicate glass (1.2 mm O.D. × 0.94 mm I.D. Harvard Apparatus, GC120TF-10) was used to pull micropipettes with resistances of 2–5 MΩ by a Sutter puller (Model P-97, Sutter Instruments), which were subsequently polished using a microforge (MF-900, Narishige). The connection between the pipette solution and the amplifier was made using 3 M KCl agar bridges. The junction potential of 16.2 mV, which was determined using the JPCalc software (P. Barry, University of South Wales, Sydney, Australia), was corrected a priori. Recordings were filtered at 5 kHz and sampled at 100 kHz using the Axopatch 200B amplifier and the Axon Digidata 1550 (Molecular Devices). pClamp 11.1 software (Molecular Devices) and Originpro 2020 (OriginLab) were used for data analysis and presentation.

### Assessment of hypertrophy in *NKX2.5*-KO organoids

To assess cellular hypertrophy, d10 *NKX2.5*-KO and control HFOs were individually dissociated into single cells and plated, and the area of eGFP-positive cells (cardiomyocytes) was determined. Dissociation was performed with the STEMdiff Cardiomyocyte Dissociation Kit (Stemcell Technologies). Single cells were resuspended in RB+ containing 10 µM Y-27632 and seeded onto a Geltrex-coated 12-well plate at one HFO per well. The plate was placed in the incubator overnight. Cells were analyzed the following day: in each well, three pictures of random spots were taken with the Axio Observer A1 (Zeiss) at ×20 magnification. Cell area was measured manually using ImageJ software. In every picture, all eGFP-positive cells were measured. Statistical analysis was performed with GraphPad Prism 7.

### Conventional cardiac differentiation of hPSCs

For 2D cardiac differentiation, 0.3 × 10^6^ HES3 NKX2.5–eGFP cells were seeded per well of a Geltrex-coated 12-well cell culture plate in 2 ml E8 medium containing 10 µM Y-27632 on d−4. Medium was exchanged on d−2 and d−1 by E8. Differentiation of cells was induced on d0 using CHIR at 7.5 µM in 3 ml RB−. After 24 h (d1), the medium was exchanged by 2 ml RB−. On d3, IWP2 was added at 5 µM in 2 ml RB− for 48 h. On d5, the medium was exchanged by 2 ml RB−. From d7 onwards, cells were kept in 2 ml RB+ per well.

For 3D cardiac differentiation, 0.5 × 10^6^ HES3 NKX2.5–eGFP cells were seeded per well of a 6-well suspension culture plate in 3 ml E8 medium containing 10 µM Y-27632 on d−2. The plate was placed on an orbital shaker. On d−1, the medium was exchanged by E8 alone. Differentiation of cells was induced on d0 using CHIR at 7.5 µM in 3 ml CDM3^31^ medium. After 24 h (d1), the medium was exchanged by 3 ml CDM3 medium containing 5 µM IWP2. From d3 onwards, cells were cultured in 3 ml CDM3 per well.

### Tissue procurement and processing of human embryonic hearts

This study was performed in accordance with institutional guidelines and was approved by the local research ethics committee (University Tübingen IRB no. 406/2011BO1). Human first-trimester hearts (*n* = 4; 5–7 weeks of gestation) were obtained from elective aborted fetuses following informed consent and de-identification. After procurement, all tissues were immediately washed in sterile Dulbecco’s PBS and processed as previously described^56^.

### RNA isolation

Before RNA isolation, single HFOs were incubated in 20 µl collagenase II (Worthington) for 30 min at 37 °C for dissociation, whereby every 5–10 min, HFOs were disrupted by pipetting up and down. A 330-µl volume of RLT buffer (Qiagen) containing 1% β-mercaptoethanol (Thermo Fisher Scientific) was added on top of the dissociated HFOs and vortexed for 15 s. Total RNA was prepared using the RNeasy Micro Kit (Qiagen) according to the manufacturer’s instructions. For RNA isolation from human embryonic hearts, cardiomyocytes from 2D cardiac differentiation and hPSCs, samples were placed into Trizol (Thermo Fisher Scientific) and mechanically disrupted by vortexing and pipetting. RNA isolation was performed using the NucleoSpin RNA Kit (Macherey-Nagel) according to the manufacturer’s instructions.

### Microarray-based mRNA expression analysis (single-color mode)

The microarray utilized in this study represents a refined version of the Whole Human Genome Oligo Microarray 4x44K v2 (Design ID 026652, Agilent Technologies), called 026652QM_RCUG_HomoSapiens (Design ID 084555) developed by the Research Core Unit Genomics of Hannover Medical School (MHH). The microarray design was created at Agilent’s eArray portal using a 1 × 1M design format for mRNA expression as a template. All non-control probes of design ID 026652 have been printed five times within a region comprising a total of 181,560 Features (170 columns × 1,068 rows). Four such regions were placed within one 1M region giving rise to four microarray fields per slide to be hybridized individually (Customer Specified Feature Layout). Control probes required for proper Feature Extraction software operation were determined and placed automatically by eArray using recommended default settings.

A 110-ng quantity of total RNA was used as input. Synthesis of Cy3-labeled antisense RNA (cRNA) was performed in three-quarters reaction volumes with the Low Input Quick Amp Labeling Kit One-Color (no. 5190-2305, Agilent Technologies) according to the manufacturer’s recommendations. cRNA fragmentation, hybridization and washing steps were carried out as recommended in the One-Color Microarray-Based Gene Expression Analysis Low Input Quick Amp Labeling Protocol V6.7, except that 2,500 ng of labeled cRNA was used for hybridization. Slides were scanned on the Agilent Micro Array Scanner G2565CA (pixel resolution 3 µm, bit depth 20). Data extraction was performed with the Feature Extraction software V10.7.3.1 by using the recommended default extraction protocol file GE1_107_Sep09.xml.

Data analysis was performed with Qlucore Omics Explorer 3.5: for statistical analysis of *NKX2.5*-KO versus control HFOs, a two-sided *t*-test was performed (*P* ≤ 0.05; fold change as indicated in the text and in Extended Data Fig. 6g). For analysis of human embryonic hearts versus HFOs, both groups were compared to hPSCs by a two-sided *t*-test (*q* ≤ 0.01; variance filtering = 0.5; fold change ≥ 10). These settings were chosen to view the 100 most significantly up/downregulated genes between the groups. For analysis of samples from 2D cardiac differentiation versus HFOs, a two-sided *t*-test was performed (*q* ≤ 0.01; fold change ≥ 10).

### scRNA-seq

HFOs were collected on d13 and individually dissociated into single cells using the STEMdiff Cardiomyocyte Dissociation Kit (Stemcell Technologies).

Library preparation for scRNA-seq analysis was performed according to the Chromium NextGEM Single Cell 3ʹ Reagent Kits v3.1 User Guide (Manual Part Number CG000204 Rev B; 10x Genomics). According to the protocol, a given excess of cells was loaded to the 10x controller to reach a target number of 10,000 cells per sample. Fragment length distribution of generated libraries was monitored using the Bioanalyzer High Sensitivity DNA Assay Kit (5067-4626; Agilent Technologies). Quantification of libraries was performed by use of the Qubit dsDNA HS Assay Kit (Q32854; Thermo Fisher Scientific).

#### Sequencing run

Libraries were pooled, denatured with NaOH, and were finally diluted accordingly to be sequenced on an Illumina NovaSeq6000 sequencer using an S4 flowcell. Samples were sequenced according to the following settings: 150 bp as sequence read 1; 150 bp as sequence read 2; 8 bp as index read 1; no index read 2. An average number of 283 million reads passing filter per sample were finally retrieved, yielding roughly 49,000 reads per cell.

#### Raw data processing

BCL files were converted to FASTQ files using bcl2fastq Conversion Software (Illumina) using the respective sample sheet with the utilized 10x barcodes. The proprietary 10x Genomics CellRanger pipeline (v3.0.2) was used with default parameters except for the setting of expected cells (--expect-cells 10,000). CellRanger was used to align read data to the reference genome provided by 10x Genomics (Human reference dataset 3.0.0; GrCh38) using the aligner STAR, counting aligned reads per gene, and calculating clustering and summary statistics. Finally, the Loupe Cell Browser from 10x Genomics was used to view and revise annotated clusters, on the basis of the implemented t-SNE algorithm.

#### Compute resource

Raw data processing was conducted on a high-performance computing cluster (HPC-seq). This cluster provides substantial compute power (600 cores in 13 nodes, a total of 5 TB RAM, 300 TB storage, Ubuntu Linux, SLURM job scheduler) to allow large workloads to be efficiently processed in a timely fashion, and furthermore provides the backbone for resource-intensive graphical applications such as the locally implemented MHH Galaxy instance. A three-tiered storage system has been implemented, with jobs transiently stored on a fast 24 TB SSD shelf, with important results saved into mid-term redundant NetApp shelves.

Further data analysis, automated *k*-means clustering and t-SNE plot generation were performed with Loupe Cell Browser 4.0.

### Statistical analysis and reproducibility

HFOs that showed the typical layered NKX2.5–eGFP pattern were considered successfully formed. All remaining HFOs (no layer formation or lack of NKX2.5–eGFP signal) were considered failed. In each experiment, 20–70 HFOs were generated, and for each experiment, the proportion of successfully formed HFOs (that is, the formation efficiency) was determined. In total, 10 independent experiments were analyzed with a total number of 418 HFOs. The mean and s.e.m. of the formation efficiencies of the 10 experiments were determined with GraphPad Prism 7.

For all following experiments, only successfully formed HFOs were used; these HFOs showed similar results in all experiments. Specifically, IF staining (whole mount, paraffin sections, cryosections), H&E staining, embedding in Geltrex or collagen I, and long-term culture experiments were repeated with at least three independent HFOs. TEM was performed on two control HFOs and one *NKX2.5*-KO organoid. Experiments with different reporter cell lines (MIXL1–GFP and *NKX2.5* KO) and the iPSC line HSC_ADCF_SeV-iPS2 were repeated at least three times with similar results. Conventional 3D cardiac differentiation experiments were performed at least three times with similar results.

All statistics were derived from at least *n* = 3 biologically independent HFOs. Statistical analysis was performed with GraphPad Prism 7 applying a two-tailed, unpaired *t*-test assuming unequal variances for the comparison of two groups. For more than two groups, a one-way analysis of variance (Tukey’s multiple comparison test) was applied. The data are presented as mean ± s.e.m. Statistical significance was assigned as not significant (NS) *P* > 0.05; *P ≤ 0.05; **P ≤ 0.01; ***P ≤ 0.001; ****P ≤ 0.0001.

### Research animals

For establishment of the anti-NFATc1 antibody staining (see Staining of paraffin sections), E11.5 embryos were isolated from CD1(ICR) mice. Mice were obtained from Charles River Laboratories. We have complied with all relevant ethical regulations and the study was approved by the Institutional Care and Use Committee, MHH (section 4 German Animal Welfare Law; animal experiment approval number 2018/200).

### Reporting Summary

Further information on research design is available in the Nature Research Reporting Summary linked to this article.

## Online content

Any methods, additional references, Nature Research reporting summaries, source data, extended data, supplementary information, acknowledgements, peer review information; details of author contributions and competing interests; and statements of data and code availability are available at 10.1038/s41587-021-00815-9.

## Supplementary information

Reporting Summary

Supplementary Video 1 SLOT of an HFO. The analysis via SLOT shows the typical layered 3D structure of HFOs.

Supplementary Video 2 Calcium imaging of an HFO with a circular beating pattern. The HFO was stained with the calcium-sensitive dye Rhod-4 to highlight the contractions.

Supplementary Video 3 Calcium imaging of an HFO with a contraction initiation point. The HFO was stained with the calcium-sensitive dye Rhod-4 to highlight the contractions.

Supplementary Video 4 MPM of an HFO. The HFO was stained with DAPI (white) and CD31 (purple) to visualize the endothelial structures. The video starts at the front of the HFO showing the IC and progresses through the ML to the OL.

Supplementary Video 5 A 3D reconstruction of the IC of an HFO. The large, branched endodermal cavities, which are distributed all over the IC of the HFO, are depicted in different colors.

Supplementary Video 6 Calcium imaging of an *NKX2.5*-KO organoid. The HFO was stained with the calcium-sensitive dye Rhod-4 to highlight the contractions.

## Data Availability

The gene expression datasets generated and analyzed during the current study are available in the Gene Expression Omnibus repository: microarray data have been deposited under accession number GSE150051 and single-cell RNA sequencing data under accession number GSE150202. For the latter, to interrogate gene expression in the t-SNE plot, the .cloupe.gz file should be downloaded, unzipped and opened with the 10x Genomics Loupe Browser. All additional data supporting the findings of this study are available within the article and its Supplementary Information.
