# Publisher Correction: Human heart-forming organoids recapitulate early heart and foregut development

**DOI:** 10.1038/s41587-021-00960-1

**Published:** 2021-05-20

**Authors:** Lika Drakhlis, Santoshi Biswanath, Clara-Milena Farr, Victoria Lupanow, Jana Teske, Katharina Ritzenhoff, Annika Franke, Felix Manstein, Emiliano Bolesani, Henning Kempf, Simone Liebscher, Katja Schenke-Layland, Jan Hegermann, Lena Nolte, Heiko Meyer, Jeanne de la Roche, Stefan Thiemann, Christian Wahl-Schott, Ulrich Martin, Robert Zweigerdt

**Affiliations:** 1 Leibniz Research Laboratories for Biotechnology and Artificial Organs (LEBAO), Department of Cardiothoracic, Transplantation and Vascular Surgery (HTTG), REBIRTH–Research Center for Translational Regenerative Medicine, Hannover Medical School, Hannover, Germany; 2 Department of Women’s Health, Research Institute for Women’s Health, Eberhard Karls University Tübingen, Tübingen, Germany; 3 The Natural and Medical Sciences Institute (NMI) at the University of Tübingen, Reutlingen, Germany; 4 Department of Medicine/Cardiology, Cardiovascular Research Laboratories, David Geffen School of Medicine at UCLA, Los Angeles, CA USA; 5 Cluster of Excellence iFIT (EXC 2180) ‘Image-Guided and Functionally Instructed Tumor Therapies’, Eberhard Karls University Tübingen, Tübingen, Germany; 6 Research Core Unit Electron Microscopy, Hannover Medical School, Hannover, Germany; 7 Institute of Functional and Applied Anatomy, Hannover Medical School, Hannover, Germany; 8 Industrial and Biomedical Optics Department, Laser Zentrum Hannover, Hannover, Germany; 9 Institute for Neurophysiology, Hannover Medical School, Hannover, Germany; 10 Biomedical Research in Endstage and Obstructive Lung Disease (BREATH), Member of the German Center for Lung Research (DZL), Hannover Medical School, Hannover, Germany; 11 Present Address: Stem Cell Discovery, Novo Nordisk A/S, Måløv, Denmark

**Keywords:** Pluripotent stem cells, Pattern formation

Correction to: *Nature Biotechnology* 10.1038/s41587-021-00815-9, published online 8 February 2021.

This paper was originally published under standard Springer Nature license (© The Author(s), under exclusive licence to Springer Nature America, Inc.). It is now available as an open-access paper under a Creative Commons Attribution 4.0 International license, © The Author(s), in accordance with the Nature Transformative Agreement for Germany. The error has been corrected in the print, HTML and PDF versions of the article.

