# Supplementary material for: Human heart-forming organoids recapitulate early heart and foregut development
Source: Nat Biotechnol. 2021 Feb 8;39(6):737–46. doi: 10.1038/s41587-021-00815-9 (PMC8192303; doi:10.1038/s41587-021-00815-9)
Supplement: Supplementary file 1 — Reporting Summary [file 41587_2021_815_MOESM1_ESM.pdf]

nature research

Corresponding author(s): Dr. Robert Zweigerdt
Lika Drakhlis

Last updated by author(s): Dec 14, 2020

Reporting Summary

Nature Research wishes to improve the reproducibility of the work that we publish. This form provides structure for consistency and transparency in reporting. For further information on Nature Research policies, see our Editorial Policies and the Editorial Policy Checklist.

Statistics

For all statistical analyses, confirm that the following items are present in the figure legend, table legend, main text, or Methods section.

n/a	Confirmed	
☐	☒	The exact sample size (*n*) for each experimental group/condition, given as a discrete number and unit of measurement
☐	☒	A statement on whether measurements were taken from distinct samples or whether the same sample was measured repeatedly
☐	☒	The statistical test(s) used AND whether they are one- or two-sided<br>*Only common tests should be described solely by name; describe more complex techniques in the Methods section.*
☒	☐	A description of all covariates tested
☐	☒	A description of any assumptions or corrections, such as tests of normality and adjustment for multiple comparisons
☐	☒	A full description of the statistical parameters including central tendency (e.g. means) or other basic estimates (e.g. regression coefficient) AND variation (e.g. standard deviation) or associated estimates of uncertainty (e.g. confidence intervals)
☐	☒	For null hypothesis testing, the test statistic (e.g. *F*, *t*, *r*) with confidence intervals, effect sizes, degrees of freedom and *P* value noted<br>*Give P values as exact values whenever suitable.*
☒	☐	For Bayesian analysis, information on the choice of priors and Markov chain Monte Carlo settings
☒	☐	For hierarchical and complex designs, identification of the appropriate level for tests and full reporting of outcomes
☒	☐	Estimates of effect sizes (e.g. Cohen's *d*, Pearson's *r*), indicating how they were calculated

Our web collection on statistics for biologists contains articles on many of the points above.

Software and code

Policy information about availability of computer code

Data collection

AxioVision SE64 Rel. 4.8
ZEN 3.0
BD Accuri C6 Software
MACSQuant Analyzer 10 Software
Feature Extraction Software V10.7.3.1

Data analysis

AxioVision SE64 Rel. 4.8
FlowJo V10
GraphPad Prism 7
ImageJ
Loupe Browser 4.0
Qlucore Omics Explorer 3.5
Reconstruct
pClamp 11.1
Originpro 2020
10x Genomics CellRanger pipeline v3.0.2

For manuscripts utilizing custom algorithms or software that are central to the research but not yet described in published literature, software must be made available to editors and reviewers. We strongly encourage code deposition in a community repository (e.g. GitHub). See the Nature Research guidelines for submitting code & software for further information.

Data

Policy information about availability of data

All manuscripts must include a data availability statement. This statement should provide the following information, where applicable:

- Accession codes, unique identifiers, or web links for publicly available datasets
- A list of figures that have associated raw data
- A description of any restrictions on data availability

The gene expression datasets generated and analyzed during the current study are available in the Gene Expression Omnibus (GEO) repository: Microarray data have been deposited under accession number GSE150051 and single-cell RNA sequencing data under accession number GSE150202. All additional data supporting the findings of this study are available within the article and its Supplementary files.

Field-specific reporting

Please select the one below that is the best fit for your research. If you are not sure, read the appropriate sections before making your selection.

☒ Life sciences ☐ Behavioural & social sciences ☐ Ecological, evolutionary & environmental sciences

For a reference copy of the document with all sections, see nature.com/documents/nr-reporting-summary-flat.pdf

Life sciences study design

All studies must disclose on these points even when the disclosure is negative.

Sample size	No sample size calculations have been performed in advance. Based on the high robustness of the HFO differentiation protocol as shown in the manuscript, for all experiments containing statistical analysis, a sample size of at least n = 3 was chosen.
Data exclusions	Not successfully formed HFOs were excluded from the analysis (see "Replication").
Replication	To determine the HFO formation efficiency, HFOs that showed the typical NKX2.5-eGFP-layered pattern were considered successfully formed. All remaining HFOs (no layer formation or lack of NKX2.5-eGFP signal) were considered failed. In each experiment, 20 – 70 HFOs were generated and for each experiment, the proportion of successfully formed HFOs (= formation efficiency) was determined. In total, 10 independent experiments were analyzed with a total number of 418 HFOs, revealing an overall efficiency of ~88%. For all following experiments, only successfully formed HFOs were used; these HFOs showed reproducible results in all experiments.
Randomization	Randomization was not relevant to this study. Only two different groups were analyzed, namely control and NKX2.5-KO HFOs. For both groups, all successfully formed HFOs were used for further analyses.
Blinding	Blinding was not relevant to this study. Only two different groups were analyzed, namely control and NKX2.5-KO HFOs. For both groups, all successfully formed HFOs were used for further analyses.

Reporting for specific materials, systems and methods

We require information from authors about some types of materials, experimental systems and methods used in many studies. Here, indicate whether each material, system or method listed is relevant to your study. If you are not sure if a list item applies to your research, read the appropriate section before selecting a response.

Materials & experimental systems

n/a	Involved in the study	
☐	☒	Antibodies
☐	☒	Eukaryotic cell lines
☒	☐	Palaeontology and archaeology
☐	☒	Animals and other organisms
☒	☐	Human research participants
☒	☐	Clinical data
☒	☐	Dual use research of concern

Methods

n/a	Involved in the study	
☒	☐	ChIP-seq
☐	☒	Flow cytometry
☒	☐	MRI-based neuroimaging

Antibodies

Antibodies used	anti-cardiac troponin T abcam #ab64623<br>anti-cardiac troponin T Thermo Fisher Scientific #MA5-12960<br>anti-CD31 Agilent #GA610<br>anti-GFP OriGene #R1091P<br>anti-HNF4α abcam #ab94748

anti-ISL1/2 DSHB #39.4D5
anti-NFAT2 abcam #ab25916
anti-NKX2.5 Cell Signaling Technology #8792
anti-(pan) myosin heavy chain (MF20) Hybridoma Bank #MF20
anti-SOX2 Cell Signaling Technology #3579
anti-SOX17 R&D systems #AF1924
anti-TBX5 Abnova #H00006910-M01
anti-vimentin abcam #ab92547
anti-WT1 Santa Cruz #sc-393498

Validation

All antibodies used were validated by the respective manufacturer. For validation of the NFAT2 antibody on paraffin sections, sections of E11.5 mouse embryos were used.

Eukaryotic cell lines

Policy information about cell lines

Cell line source(s)

Human embryonic stem cell lines:
HES3 NKX2.5-eGFP: Obtained from S. C. Den Hartogh and R. Passier (Department of Anatomy and Embryology, Leiden University Medical Centre)
HES3 MIXL1-GFP: Obtained from E. G. Stanley and A. G. Elefanty (Monash Immunology and Stem Cell Laboratories, Monash University)
HES3 NKX2.5-eGFP/eGFP: Obtained from D. J. Anderson and D. A. Elliott (Murdoch Children's Research Institute, Royal Children's Hospital)

Human induced pluripotent stem cell line:
HSC_ADCF_SeV-iPS2103: Obtained from A. Haase (Hannover Medical School)

Authentication

All applied cell lines are well-established and published:
HES3 NKX2.5-eGFP: Elliott et al., Nature Methods, 2011; Den Hartogh et al., Stem Cells, 2015
HES3 MIXL1-GFP: Davis et al., Blood, 2007
HES3 NKX2.5-eGFP/eGFP: Anderson et al., Nature Communications, 2018
HSC_ADCF_SeV-iPS2103: Haase et al., Stem Cell Research, 2017

Mycoplasma contamination

All cell lines were routinely tested for myocaplasma contamination and were tested negative.

Commonly misidentified lines
(See ICLAC register)

No such lines have been used.

Animals and other organisms

Policy information about studies involving animals; ARRIVE guidelines recommended for reporting animal research

Laboratory animals

E11.5 mouse embryos from CD1(ICR) mice

Wild animals

The study did not involve wild animals.

Field-collected samples

The study did not involve samples collected from the field.

Ethics oversight

The study was approved by the Institutional Care and Use Committee, Hannover Medical School (§4 German Animal Welfare Law; animal experiment approval number 2018/200).

Note that full information on the approval of the study protocol must also be provided in the manuscript.

Flow Cytometry

Plots

Confirm that:

☒ The axis labels state the marker and fluorochrome used (e.g. CD4-FITC).

☒ The axis scales are clearly visible. Include numbers along axes only for bottom left plot of group (a 'group' is an analysis of identical markers).

☒ All plots are contour plots with outliers or pseudocolor plots.

☒ A numerical value for number of cells or percentage (with statistics) is provided.

Methodology

Sample preparation

HFOs were individually dissociated into single cells using the STEMdiff Cardiomyocyte Dissociation Kit (Stemcell Technologies) and stained by applying the Fix&Perm kit (Nordic MUbio).

Instrument

Cells were measured at a BD Accuri C6 flow cytometer or at a MACSQuant Analyzer 10 (Miltenyi Biotec).

Software	Data were analyzed with FlowJo V10.
Cell population abundance	No sorting was performed in this study.
Gating strategy	Living cells were gated within the FSC/SSC plot following doublet exclusion. Unstained samples were used to distinguish between negative and positive signals of antibodies.

☒ Tick this box to confirm that a figure exemplifying the gating strategy is provided in the Supplementary Information.
